# UbFluor: a mechanism-based probe for HECT E3 ligases†
†Electronic supplementary information (ESI) available. See DOI: 10.1039/c6sc01167e


**DOI:** 10.1039/c6sc01167e

**Published:** 2016-05-17

**Authors:** David T. Krist, Sungjin Park, Galyah H. Boneh, Sarah E. Rice, Alexander V. Statsyuk

**Affiliations:** a Department of Chemistry , Center for Molecular Innovation and Drug Discovery , Chemistry of Life Processes Institute , Northwestern University , Silverman Hall, 2145 Sheridan Road , Evanston , Illinois 60208 , USA . Email: a-statsyuk@northwestern.edu; b Department of Cell and Molecular Biology , Feinberg School of Medicine , Northwestern University , 303 East Chicago Avenue , Chicago , Illinois 60611 , USA

## Abstract

Homologues to E6AP Carboxyl Terminus E3 ubiquitin ligases (HECT, ∼28 known) are genetically implicated in cancer, neurological, hypertensive, and autoimmune disorders, and are potential drug targets to treat these diseases. The major bottleneck in the field of HECT E3s is a lack of simple assays to quantify the enzymatic activity of these enzymes in the presence of small molecules. Typical assays require E1, E2, HECT E3, ubiquitin (Ub), ATP and additional reagents to detect the resulting free poly-ubiquitin chains. To address this need, we developed UbFluor, a fluorescent thioester conjugate between the C-terminus of Ub and fluorescein-thiol (Fluor-SH). UbFluor is a mechanism-based probe that undergoes a direct transthiolation reaction with the catalytic cysteine of the model HECT E3 ligase Rsp5, producing the catalytically active Rsp5∼Ub (∼ indicates thioester) accompanied by release of Fluor-SH. The kinetics of this two-component reaction can be easily monitored with real-time fluorescence polarization (FP) assays. Importantly, UbFluor eliminates the need to use SDS-PAGE, ATP, E1 and E2 enzymes, and extra poly-ubiquitin chain detection reagents. Although the developed system lacks ATP, and E1 and E2 enzymes, we show that UbFluor can recapitulate the native ubiquitination reaction by detecting and quantifying defects in transthiolation and isopeptide ligation of Rsp5 HECT E3 alanine mutants. Based on our findings, we show that UbFluor can be utilized to conduct high-throughput screens (HTS) of small molecules against HECT ligases.

## Abstract 2

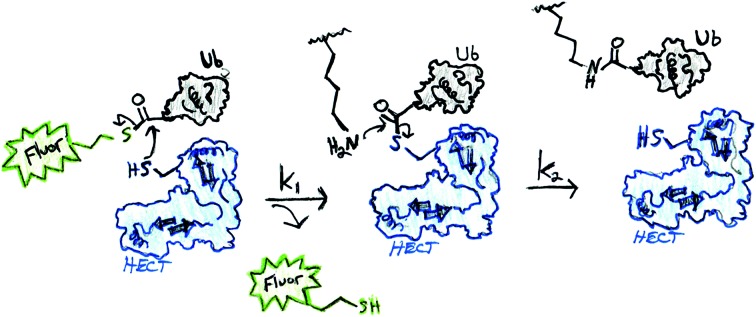
UbFluor is a mechanism-based probe that undergoes a direct transthiolation reaction with the catalytic cysteine of the model HECT E3 ligase Rsp5. We show that UbFluor can be utilized to conduct high-throughput screens (HTS) of small molecules against HECT ligases.

## Introduction

Approximately 800 ubiquitin enzymes (E1, E2, and E3 ligases and deubiquitinating enzymes) regulate the dynamic ubiquitination of ∼19 000 protein substrates, thus representing a vast and unexplored area of the human druggable genome.^1^ Among these, HECT E3 family ligases (∼28 known) have been genetically implicated in autism,^2^ Angelman syndrome,^3^ Liddle's syndrome,^4^ cancers,^5^ and autoimmune disorders,^6^ thus highlighting their significance in human biology and medicine.

A major challenge in studying the biochemistry of HECT E3s and discovering chemical probes for this family of enzymes is the complexity and the speed of the ubiquitination reaction.^7^ Typically, five components are required for an *in vitro* reaction (ATP, Ub, E1, E2, and the HECT E3 enzyme), which generates complex mixtures of E1∼Ub, E2∼Ub, and HECT∼Ub thioesters, free poly-ubiquitin chains, and auto-ubiquitinated E3 ligase.^8^ Furthermore, radioactive ubiquitin and labour-intensive SDS-PAGE gels are often used to quantify and separate the various Ub thioesters and poly-ubiquitinated substrates.^9^ Overall, such complexity is a major bottleneck that makes it difficult to quantify changes in the enzymatic activity of E3 ligases upon biochemical point mutation or small molecule inhibition. Furthermore, this complexity makes it difficult to conduct large-scale HTS, which are prone to false positives from off-target inhibition of E1 and E2 enzymes and their respective thioesters.^10^

Recently, we discovered an E2 enzyme-independent “bypassing system” (ByS) in which the catalytic cysteine of HECT E3 undergoes a direct transthiolation reaction with the C-terminal ubiquitin thioester Ub∼MES (mercaptoethanesulfonate) producing the catalytically active HECT E3∼Ub intermediate.^11^ The resulting HECT E3∼Ub thioester can undergo auto-ubiquitination, ligate ubiquitin onto a substrate, and build poly-ubiquitin chains. However, quantification of Ub∼MES consumption using native chemical ligation requires quenching of a reaction mixture followed by labour intensive SDS-PAGE analysis, which complicates kinetic analysis and introduces a large degree of measurement error, precluding its widespread use for HTS or quantitative biochemical studies.

**Fig. 1:**
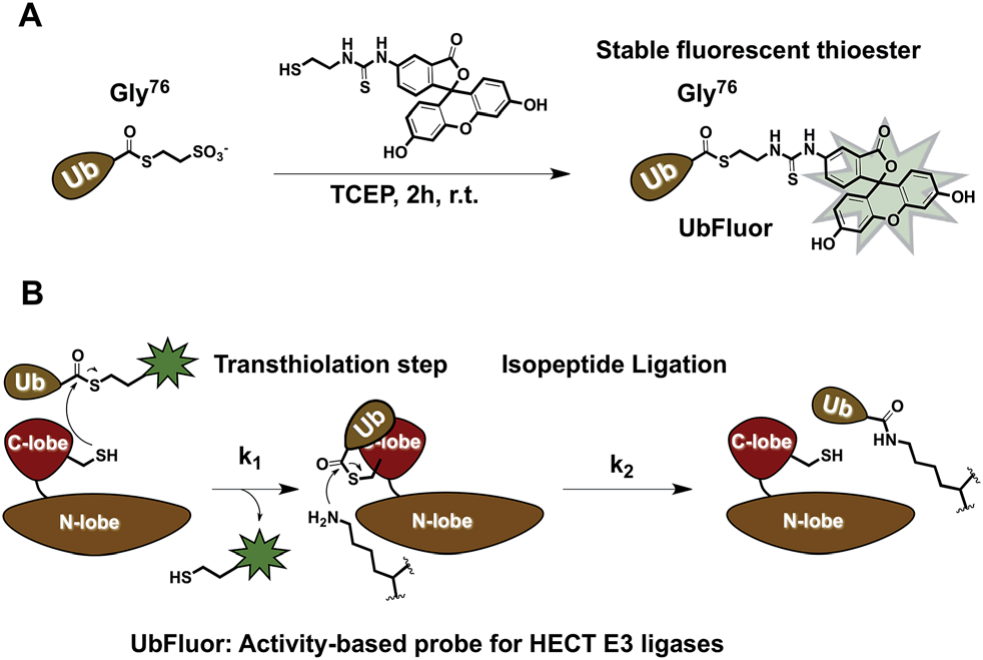
(A) Synthesis of UbFluor. (B) UbFluor reacts with HECT ligase through transthiolation to produce the HECT∼Ub thioester while liberating Fluor-SH. The catalytic domain of HECT E3 is composed of a C-lobe and N-lobe linked *via* a flexible hinge region. Clearance of the HECT E3∼Ub thioester can be accomplished through isopeptide ligation. Even though Fluor-SH is liberated through transthiolation, we can still detect *k*_2_ when isopeptide ligation defects are introduced to the HECT domain.

To overcome these limitations, we developed UbFluor, a novel mechanism-based probe for HECT E3s with built-in fluorescence polarization read-out of enzyme activity in real-time (Fig. 1A). This probe allows direct and quantitative measurements of HECT E3 activity in the absence of ATP, and E1 and E2 enzymes. UbFluor features Fluor-SH conjugated to the C-terminal Gly^76^ of ubiquitin *via* a thioester linkage. We show that the catalytic cysteine of the model HECT E3 ligase Rsp5 undergoes transthiolation with the UbFluor thioester to liberate Fluor-SH, and to generate the catalytically active Rsp5∼Ub thioester. Our data suggest that UbFluor engages the same surface of the Rsp5 HECT C-lobe that binds the Ub of the native E2∼Ub thioester for the subsequent transthiolation. This transthiolation step can be monitored using FP to directly observe the consumption of UbFluor in real-time in a 384-well plate without adding other reagents (Fig. 1B).

Moreover, by simply changing the ratio of HECT E3 : UbFluor, reactions can be run under single turnover conditions (ST, excess of HECT E3) to measure transthiolation rates (*k*_1_), or under multiturnover conditions (MT, excess of UbFluor) to observe the overall rate of UbFluor turnover, which includes both transthiolation and clearance of the HECT∼Ub thioester (steps *k*_1_ and *k*_2_, Fig. 1B). This allows a simple decoupling of the functional roles of HECT E3 residues involved in transthiolation or isopeptide ligation steps. With ST, we approximate an environment in which each ligase molecule reacts with at most one UbFluor molecule. Therefore, the observed reaction rates directly assess the transthiolation of UbFluor by the HECT catalytic cysteine (*k*_1_). However, under MT conditions, we observe an environment in which each ligase molecule has the opportunity to process more than one UbFluor molecule. Any defect that prevents the discharge of Ub from the HECT∼Ub thioester will limit UbFluor consumption because the HECT catalytic cysteine is unable to react with another molecule of UbFluor. For example, if there is a defect in the isopeptide ligation step (*k*_2_) due to small molecule inhibition or a biochemical point mutation then there will be an accumulation of inactive HECT E3∼Ub thioester. This inactive thioester will not be able to react with another molecule of UbFluor.

We first demonstrate that the reaction of UbFluor with Rsp5 HECT E3 is relevant to the native ubiquitination cascade, thus validating its use as a tool to study the biochemistry of HECT E3s and to discover chemical probes. Analysing the reaction between UbFluor and each of the previously reported 18 Rsp5 HECT alanine point mutants under ST and MT conditions,^12^ we show that UbFluor detects and quantifies known defects in both native Rsp5 transthiolation and native isopeptide ligation steps. Although the reaction with UbFluor is E2 enzyme-independent, our studies show that the reaction of UbFluor with Rsp5 otherwise recapitulates a native ubiquitination reaction such that UbFluor is a suitable probe for (1) discovery of residues relevant to HECT E3 catalysis involving transthiolation and isopeptide ligation steps, and (2) HTS to discover chemical probes for HECT E3s. As an example, we used UbFluor to discover catalytically relevant residues of Rsp5 and to introduce a UbFluor HTS assay to discover small molecule probes of HECT E3s.

## Results

### 1. Synthesis of UbFluor

To make UbFluor we subjected Ub∼MES to a large excess of Fluor-SH (Fig. 1A, ESI†). Transthiolation of Ub∼MES to yield UbFluor proceeded efficiently with a 60% yield following purification by size-exclusion chromatography (ESI†).

### 2. Reaction of UbFluor with ΔWW Rsp5

For our initial experiments, we utilized the well-studied *S. cerevisiae* HECT E3 ligase Rsp5. Rsp5 is an essential enzyme in *S. cerevisiae* and is the orthologue of human Nedd4-1, which is implicated in mitotic chromatin assembly,^13^ ribosome stability,^14^ and protein trafficking.^15^ Rsp5 harbours an N-terminal C2 domain, followed by three WW domains and a C-terminal catalytic HECT domain (Fig. S1†).^16^,^17^

**Fig. 2:**
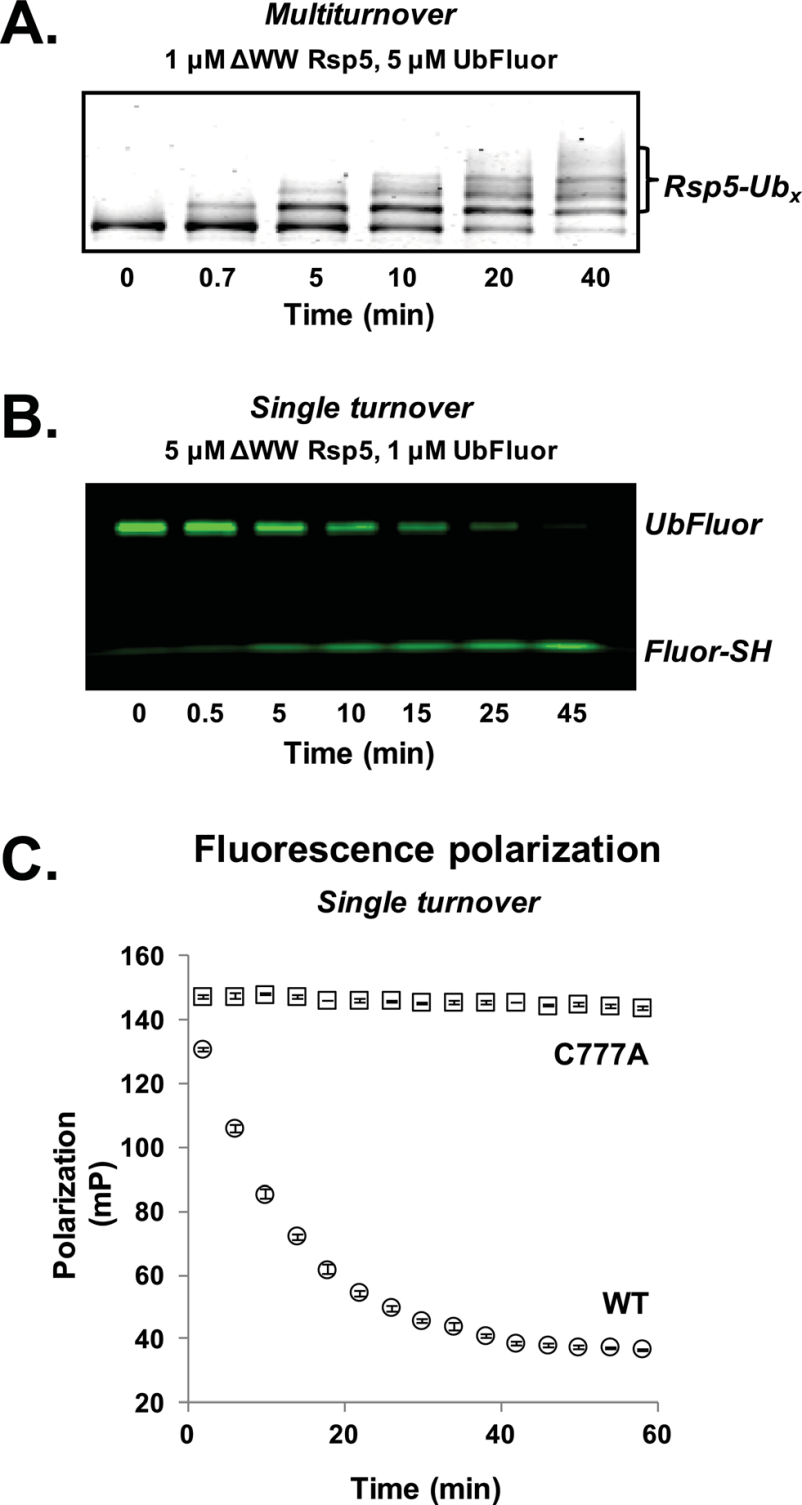
(A) Gel-based analysis of reactions between UbFluor and ΔWW Rsp5 under MT reaction conditions (Sypro orange stain); (B) ST reaction conditions (fluorescence scanning). (C) FP of reactions with UbFluor (1 μM) and wild type ΔWW Rsp5 (5 μM, circles) or catalytically inactive ΔWW Rsp5 C777A (5 μM, squares) in a 384-well plate. Mean ± SEM for 3 separate reaction trials are shown. Data from every 4 minutes are plotted.

Initially, we prepared a previously used ΔWW Rsp5 construct that lacks the three WW domains and auto-ubiquitinates in the presence of excess UbFluor (Fig. S1,† residues 231–420 removed from the full length protein).^11^ Because this construct auto-ubiquitinates under MT conditions, we readily observe auto-ubiquitinated ligase as the product of isopeptide ligation (Fig. 2A, S2 and S3†).

To first confirm that UbFluor undergoes transthiolation with the catalytic cysteine of the HECT domain, we treated UbFluor (1 μM) with an excess of wild-type (WT, 5 μM) or catalytically inactive ΔWW Rsp5 C777A (5 μM) under ST conditions. A time-course analysis of these reactions with non-reducing SDS-PAGE gels showed diminution of the UbFluor band with the simultaneous liberation of Fluor-SH for WT ΔWW Rsp5 but not for ΔWW Rsp5 C777A (Fig. 2B and S2†).

### 3. UbFluor is a suitable probe for real-time fluorescence polarization kinetics

As UbFluor (MW 9013 Da) liberates Fluor-SH (MW 467 Da) upon transthiolation, the associated change in fluorescence polarization can be monitored in real-time using a plate reader to obviate the need for gel-based analysis. Both ST and MT reactions can be performed in a 384-well plate (20 μL or less per well) and monitored with kinetic FP (Fig. 2C and S2†). To convert polarization values (mP) to units of UbFluor concentration (μM), raw polarization data was converted according to a linear relationship observed between polarization and the ratio of UbFluor to free Fluor-SH and Ub (Fig. S4 and Table S1†).

We observed that the pool of available UbFluor was consumed more rapidly under ST reaction conditions (Fig. S2†), and that initial velocities of consumption varied linearly with enzyme concentration (Fig. S5†). The gel-based and FP measurements of UbFluor consumption were in agreement under both ST and MT reaction conditions (Fig. S2†). We closely examined the FP signal from UbFluor reactions to evaluate any changes that do not result from transthiolation with Rsp5. In all of our MT measurements, we observed an initial slight decrease in UbFluor polarization even in the absence of the Rsp5 enzyme, or in the presence of the catalytically inactive Rsp5 C777A mutant (Fig. S2†). Such FP signal decay has been observed in other types of FP assays; however at this stage, we cannot provide a definitive explanation of why this apparent decrease in signal occurs.^18^ This “background” UbFluor consumption has a maximal change of ∼15 mP (14% of the total dynamic range), but when taken into account does not affect the overall conclusions of this work.

Another concern that arose during our studies was the significant fluorescence quenching that occurred at UbFluor concentrations greater than 200 μM that could possibly affect our FP measurements. However, this quenching appeared to diminish the parallel and perpendicular fluorescence intensities to a comparable degree. Since fluorescence polarization is a ratiometric value involving parallel and perpendicular fluorescence intensities [FP = (*F*_∥_ – *F*_⊥_)/(*F*_∥_ + *F*_⊥_)], FP should remain reliable if the parallel and perpendicular intensity quenching offset each other (Fig. S6A†).

To test the reliability of FP measurements in the quenching regime, we mixed several ratios of UbFluor to Ub + Fluor-SH in the presence of catalytically inactive Rsp5 HECT C777A and measured FP. With 400 μM total fluorophore in each of the measured solutions, we observed a linear correlation between polarization and the ratio of UbFluor to Ub + Fluor-SH (Fig. S6B†). Therefore, fluorescence polarization can reliably monitor UbFluor consumption up to at least 400 μM fluorophore.

Additionally, during UbFluor reactions the liberated Fluor-SH could potentially bind the ligase and influence subsequent catalysis. To avoid disulfide formation with the catalytic or surface cysteines, we included the reducing agent TCEP (0.5 mM) in our assays. Additionally, we avoid non-covalent Rsp5-Fluor-SH interactions by adding the non-ionic detergent Tween-20 to our reactions (6 μM, 10% of the critical micelle concentration). To examine the influence of free Fluor-SH on Rsp5 catalysis, we measured the amount of ΔWW Rsp5 auto-ubiquitination in the presence of increasing amounts of Fluor-SH. We only started to see dose-dependent inhibition of the reaction of ΔWW Rsp5 (1 μM) with UbFluor (10 μM) in the presence of 80 μM free Fluor-SH (Fig. S6C†). Since our reaction conditions contain 1–5 μM Rsp5 and 0.25–20 μM UbFluor, these results suggest that the effect of liberated Fluor-SH on Rsp5 is negligible.

### 4. Michaelis–Menten kinetics reveal a high *K*_M_ for Ub-Fluor/HECT domain

For kinetic studies, rather than using the ΔWW Rsp5 construct that auto-ubiquitinates, we used the isolated catalytic HECT domain of Rsp5 (Fig. S1,† residues 421-809, referred to as Rsp5 HECT). Following reaction of Rsp5 HECT with UbFluor, the generated HECT∼Ub thioester does not auto-ubiquitinate but rather produces Ub-UbFluor (di-UbFluor) by ligating Ub to a lysine of UbFluor (Fig. S3†). This approach eliminates the challenge of producing enzymatically active Rsp5·Ub_*x*_ adducts, which would complicate the calculation and understanding of *k*_cat_ and *K*_M_ values. To initially define the enzymatic relationship between UbFluor and HECT ligase, we determined the Michaelis–Menten parameters of the MT consumption of UbFluor by Rsp5 HECT. Initial reaction velocities at several concentrations of UbFluor were measured for the first 15 minutes where linearity is indicated by *R*^2^ > 0.98 and less than 10% of UbFluor has been consumed. Thus, by treating the Rsp5 HECT domain with an excess of UbFluor we measured an apparent *k*_cat_ of 0.094 ± 0.010 s^–1^ and an apparent *K*_M_ of 791.9 ± 121 μM (Fig. S6D and E†).

### 5. Pseudo first-order determination of bimolecular rates allows rapid enzyme characterization

**Fig. 3:**
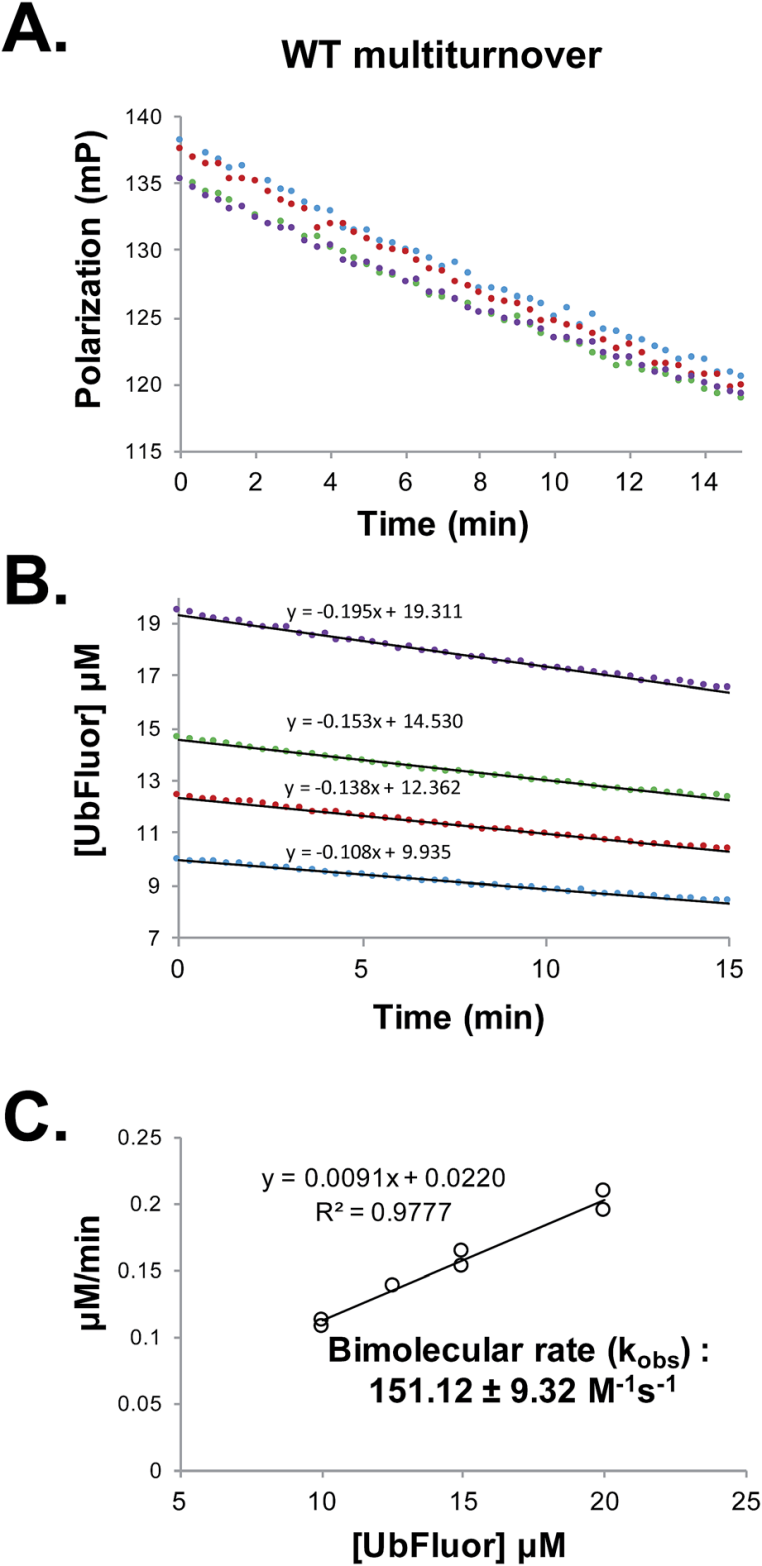
(A) Raw polarization data from a multiturnover experiment with WT Rsp5 HECT : Rsp5 HECT (1 μM) with UbFluor (10 μM (blue), 12.5 μM (red), 15 μM (green), 20 μM (purple)). Measurements were taken every 20 seconds for 15 minutes. (B) Polarization values from (A) are converted to concentrations of UbFluor according to Table S1.† Linear trendline fits are given. (C) Slopes from the lines in (B) and from a replicate experiment performed at the same enzyme and UbFluor concentrations are plotted against UbFluor concentrations (8 total measurements). The resulting slope of the line (*k*_obs_ = 0.0091 min^–1^) is converted into the bimolecular rate by dividing by enzyme concentration and by 60 s min^–1^. Error: ±error in the slope.

Determining Michaelis–Menten constants requires a prohibitively large amount of UbFluor and is thus not amenable to the kinetic evaluation of many mutants. However, kinetic parameters can also be evaluated under pseudo first-order conditions to obtain an apparent bimolecular rate constant *k*_obs_ (M^–1^ s^–1^) in which rate = *k*_obs_[enzyme][UbFluor]. With sub-saturating UbFluor concentrations far below *K*_M_, this constant can be calculated under ST or MT conditions. Thus, we determined ST bimolecular rates by measuring the initial velocities of reactions with 5 μM Rsp5 HECT with 0.25, 0.50, 0.75, and 1.00 μM UbFluor (Fig. S7†). MT bimolecular rates were determined using 1 μM Rsp5 HECT with 10, 12.5, 15, and 20 μM UbFluor (Fig. 3 and S8†). With these conditions, we were confident that UbFluor could be used to quantitatively assess residue-specific contributions to HECT E3 ligase catalysis. In both cases there was a linear relationship between the rates and UbFluor concentration. Thus, the activities of HECT mutants can be compared according to apparent bimolecular rates (*k*_obs_), which contain data from four separate UbFluor concentrations.

We are aware that using bimolecular rates to compare the activity of mutant to wild-type enzymes can be misleading if the ratio of the mutant to wild-type reaction rates (*V*_mut_/*V*_WT_) depends on the substrate concentration. However, for the bi-molecular rates that we report, *V*_mut_/*V*_WT_ is invariant across the range of UbFluor concentrations tested (0–20 μM, Fig. S9†).^19^ As a control, we also calculated bimolecular rates of the reaction of the Rsp5 C777A mutant with UbFluor (Fig. S7 and S8†). The low background reaction rates observed with C777A are due to the initial loss of fluorescence polarization as was discussed earlier. Thus, by determining the bimolecular rate constants of specific HECT point mutants under ST or MT reaction conditions, we can identify residues that (1) affect UbFluor transthiolation (*k*_1_), and that (2) impact the mechanisms following transthiolation, such as isopeptide ligation steps (*k*_2_).

### 6. The native cascade and UbFluor reactions report similar enzymatic defects for a set of Rsp5 mutants

To be useful, UbFluor should quantitatively report changes in Rsp5 activity relevant to the native ubiquitination cascade that are caused by biochemical mutations or small molecules. Previously, we demonstrated that E2-independent ubiquitination recapitulates the native ligation mechanism following formation of the E3∼Ub thioester.^11^ However, proper interpretation of UbFluor assays requires that we understand how UbFluor transthiolation differs from native E2∼Ub transthiolation. To do so, ST analysis was used to isolate the mechanism of UbFluor transthiolation, while MT assays were used to measure the total processing of UbFluor through transthiolation and isopeptide ligation steps. Importantly, the MT UbFluor assays are most relevant to Rsp5 activity changes in the native cascade where mutation or small molecule modulation impacts the ability of HECT ligases to transfer Ub from the E2∼Ub thioester to the substrate.

**Fig. 4:**
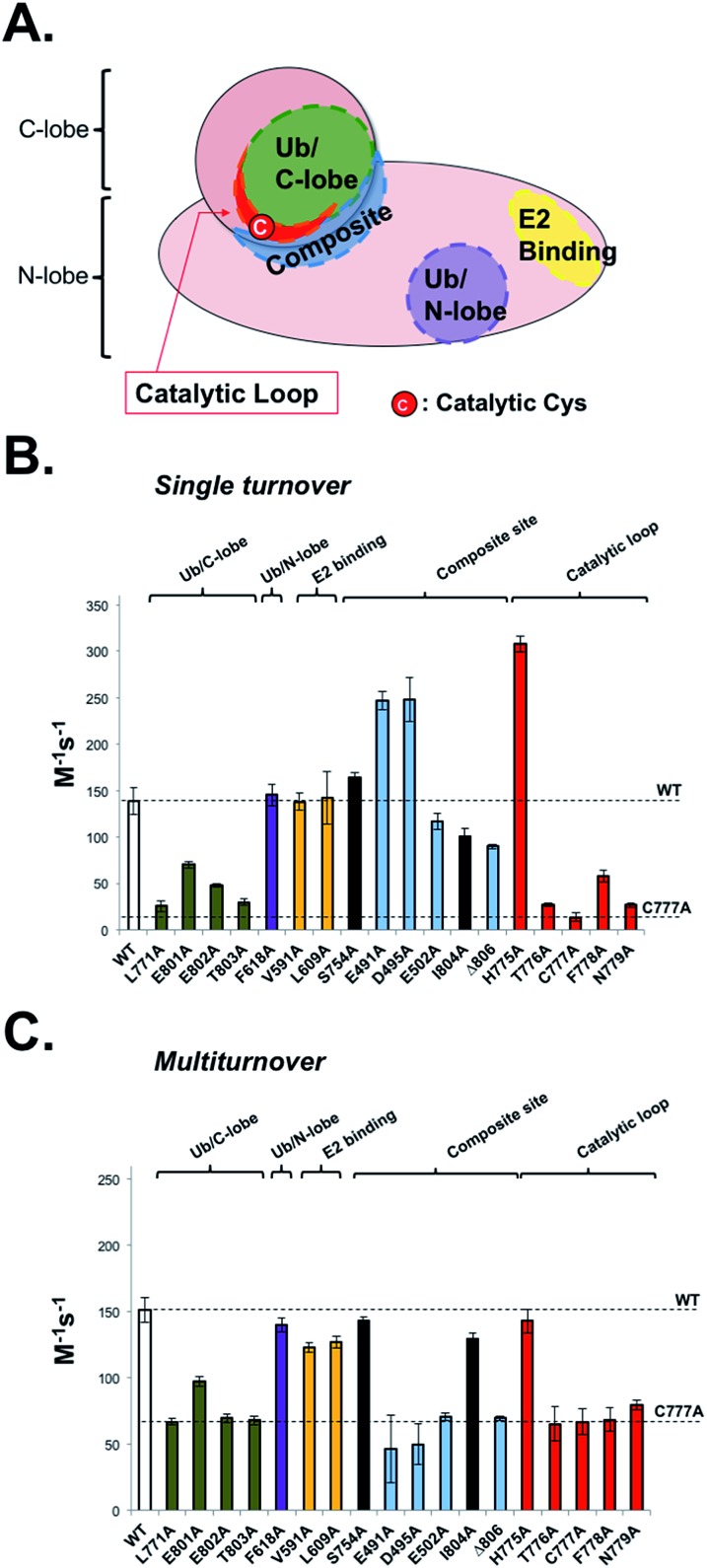
UbFluor detects biochemical defects in HECT E3 ligases. (A) Representative map of biochemical point mutations throughout the catalytic HECT domain of Rsp5. (B) Rsp5 HECT (5 μM) and its corresponding mutants were treated with UbFluor (0.25, 0.50, 0.75, and 1.00 μM) under ST reaction conditions. (C) Rsp5 HECT (1 μM) and its corresponding mutants were treated with UbFluor (10.0, 12.5, 15.0, and 20.0 μM) under MT reaction conditions. In both (B) and (C), enzyme efficiency is represented as the bimolecular rate (M^–1^ s^–1^) as described in Fig. S7 and S8.† The mutations corresponding to black bars are not classified to a certain region and were not previously seen to disrupt Rsp5 catalysis. The error bars for each mutant are obtained from linear fits of at least two repeats of UbFluor consumption at four different concentrations (8 total measurements per mutant).

We purified 15 alanine point mutants of the Rsp5 HECT domain that are defective in either transthiolation with E2∼Ub or in native isopeptide ligation steps as was reported previously (Fig. 4A).^12^ Furthermore, we purified 2 alanine point mutants that were not previously investigated (N779A and I804A), and one alanine point mutant that did not markedly affect the catalysis in a previous study (S754A).^12^ We classified all mutated residues into 5 groups according to their location and known roles in Rsp5 catalysis: (1) Ub/C-lobe interface: L771, E801, E802, and T803, (2) Ub/N-lobe interface: F618, (3) E2 binding site: V591 and L609, (4) composite isopeptide ligation site: S754, E491, D495, E502, I804, and Δ806 (stop codon at F806), and (5) the catalytic loop near the catalytic Cys^777^: H775, T776, C777, F778, and N779. The Ub/C-lobe interface binds Ub of E2∼Ub and is essential for E2∼Ub transthiolation,^20^ as well as for ligation.^21^ However, the Ub/N-lobe interface is important for efficient poly-Ub chain formation,^22^–^24^ autoinhibition,^25^–^27^ and additional modulatory roles.^28^ The E2 binding site is essential for the HECT domain to bind E2∼Ub.^20^ The composite site is the collection of residues that participate in the formation of the transient catalytic architecture between C- and N-lobes that is essential for efficient ligation of Ub onto the substrate lysine.^12^ Finally, the catalytic loop on the C-lobe harbours the catalytic cysteine (Cys^777^) in addition to the flanking residues that have poorly defined roles in E2∼Ub transthiolation and ubiquitin ligation mechanisms.

Because UbFluor does not use the E2 enzyme, we expect that the molecular details of UbFluor transthiolation by Rsp5 Cys^777^ may differ from the native mechanism of E2∼Ub transthiolation. We hypothesized that UbFluor transthiolation should depend on one of the two ubiquitin interacting surfaces in the HECT domain, but not the E2 binding site. To be useful, UbFluor transthiolation should exploit the C-lobe (but not the N-lobe) ubiquitin binding surface of the catalytic HECT domain that is essential for the native transthiolation between E2∼Ub and HECT E3.^20^ To find out which (if any) HECT E3 surface is critical for UbFluor transthiolation, we investigated Rsp5 HECT with mutations on either the C- or N-lobe binding surfaces under ST conditions (Fig. 4B). Only mutations on the C-lobe interface significantly lowered the ST rate with UbFluor, while the F618A mutation that disrupts the binding of ubiquitin at the N-lobe site did not impact the ST rate (Fig. 4B). These results indicate that UbFluor utilizes the C-lobe ubiquitin binding surface to undergo transthiolation, similar to the native transthiolation reaction with E2∼Ub.^20^ Since the F618A mutant performed similar to WT under MT conditions, the ubiquitin binding site at the N-lobe of the Rsp5 HECT domain may not be a binding site for acceptor ubiquitin,^28^ even though this site is important for ligase processivity.^42^ As expected, mutating the E2 binding site, which causes significant defects in native transthiolation with E2∼Ub, did not affect the UbFluor ST rate.^12^,^29^ It is noteworthy that although UbFluor encounters the C-lobe ubiquitin binding surface to undergo transthiolation just as in the native reaction, the UbFluor assay cannot detect defects in E2 binding because of its unique E2-independent transthiolation mechanism.

We did not see a substantial defect in the ST rates for two composite site mutations (E502A and Δ806), while both were defective in isopeptide bond ligation under MT (Fig. 4B and C). This is expected since these mutations were previously reported to impede ligation of HECT∼Ub onto the substrate lysine but not transthiolation of E2∼Ub.^11^,^12^,^30^ It was previously suggested that the E502A mutation disrupts the isopeptide ligation architecture of Rsp5, thus impeding the ligation step.^12^ Notably, two composite site mutations (E491A and D495A) enhanced UbFluor transthiolation rates although they were not seen to affect native E2∼Ub transthiolation rates.^12^ Despite this unexpected activation of UbFluor transthiolation by the E491A and D495A mutants, their MT rates are significantly decreased compared to WT, which coincides with the activity defects of those mutants in the native isopeptide ligation reaction (Fig. 4C).^12^ Notably, the residue equivalent to E491 in E6AP is a known Angelman syndrome mutation.^2^ Since the intent is to use UbFluor in MT assays to assess overall enzymatic activity of E3 enzymes for HTS and biochemical purposes, the enhanced transthiolation rates of UbFluor with E491A and D495A Rsp5 mutants under ST do not undermine that purpose. As E491A, D495A, E502A and Δ806 Rsp5 mutants are defective in isopeptide ligation, we confirmed that the E491A and Δ806 mutants did not produce di-ubiquitin as assessed by SDS-PAGE under ByS reaction conditions (Fig. S10 and S11A†).

The T776A, C777A and F778A mutations on the catalytic loop are known to cause defects in native transthiolation.^12^ We confirmed this same trend under UbFluor ST and MT analysis (Fig. 4B and C). The Rsp5 N779 residue was not previously characterized, but is also conserved across the Nedd4 family of HECT E3s in addition to the majority of other HECT E3s (22 of the 28 known, Table S2†). Interestingly, we found Rsp5 N779A to be defective in UbFluor ST and MT analysis (Fig. 4B and C), and then further determined this mutant to be defective in performing transthiolation of UbcH5B∼Ub according to a gel-based assay (Fig. S11B†). The role of N779 in Rsp5 catalysis was not previously known. Thus, UbFluor can be used to discover residues on HECT E3s that are important for catalysis.

Unexpectedly, Rsp5 H775A showed enhanced UbFluor transthiolation under ST despite demonstrating defective native transthiolation with its cognate E2∼Ub, UbcH5B∼Ub (Fig. 4B and S11B†). However, H775A Rsp5 was as active as wild-type under MT conditions (Fig. 4C and S11A†). A previous co-crystal structure of Nedd4L bound to the UbcH5B∼Ub oxyester suggests that the residue equivalent to His^775^ mediates specific E2-HECT interactions (Fig. S12†).^20^ However, since UbFluor has fluorescein in place of UbcH5B (a cognate E2 in the native reaction), we thought that UbFluor might simulate a non-cognate E2∼Ub thioester. Therefore, one functional role of His^775^ in Rsp5 could be to protect Rsp5 from transthiolation with non-cognate E2∼Ub thioesters, and to promote transthiolation with cognate E2∼Ub thioesters. Our data from native ubiquitination cascade reactions support this possibility (Fig. S13†).

Taken together, our results suggest that UbFluor can detect and quantify native biochemical defects in transthiolation and isopeptide ligation steps of HECT E3s, and therefore can be used in HTS assays and biochemical studies. However, caution is needed when interpreting UbFluor data (especially regarding the transthiolation step) since it utilizes an E2-independent transthiolation mechanism that is similar but not identical to the native reaction.

### 7. HECT E3∼Ub thioesters demonstrate distinct reactivity to lysine

During our studies we examined the effect of lysine on ST and MT reaction rates to verify that MT conditions uniquely measure ligation. Because lysine can act as a Ub acceptor, it can enhance clearance of the HECT∼Ub thioester, and thus the overall rate of the MT reaction. Therefore, the addition of lysine should increase the rate in the ligation (*k*_2_) step, facilitating HECT E3∼Ub thioester clearance and the regeneration of free HECT E3. The addition of lysine should only enhance the MT rate, but not the ST rate. For our studies we used Rsp5 HECT E491A, Rsp5 HECT D495A, Rsp5 HECT E502A and Rsp5 HECT Δ806, which are defective in isopeptide ligation (Fig. 4C). 12,31

**Fig. 5:**
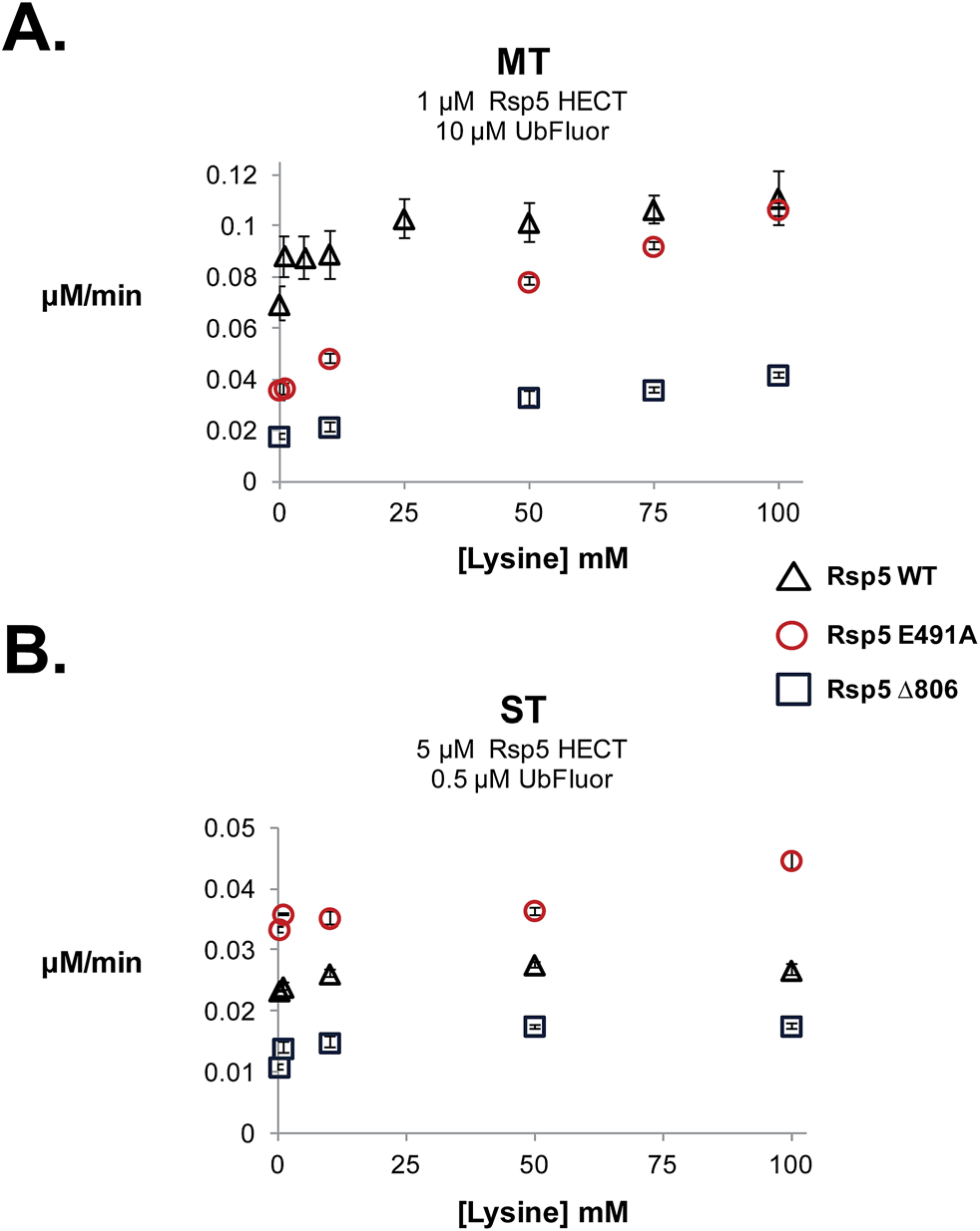
(A) MT initial velocities were measured with fluorescence polarization. Reactions were run in 150 mM NaCl, 0.5 mM TCEP, 6 μM Tween-20, 50 mM HEPES pH 7.5 with the indicated amount of l-lysine. The indicated amount of l-lysine was added as part of a 10× buffer (pH was then adjusted to 7.5 for each 10× buffer). (B) ST initial velocities were measured with fluorescence polarization under the same buffer conditions described for (A). A background subtraction of raw polarization data was performed based on reactions without ligase at each concentration of l-lysine for (A) and (B). The plotted data are mean ± SEM for 3 separate reaction trials.

It was previously suggested that Glu^491^ of Rsp5 forms part of an acidic loop that is critical for the stabilization of the ligation conformation.^12^ Consequently, Rsp5 E491A is defective in native cascade isopeptide ligation and in the UbFluor MT assay (Fig. 4C). When lysine was added to the UbFluor reaction with Rsp5 HECT E491A, MT reaction rates increased in a dose-dependent manner reaching a 3-fold rate increase in the presence of 100 mM l-lysine, effectively reaching the activity of WT Rsp5 HECT under the same reaction conditions (Fig. 5A). Under single turnover conditions, however, Rsp5 E491A is relatively unresponsive to up to 50 mM lysine (Fig. 5B). Further confirming our observations, we found that accumulated Rsp5 E491A∼Ub thioester can be consumed by lysine or β-mercaptoethanol (β-ME), but not by l-arginine (Fig. S14A–D†). Alternatively, Rsp5 ligation can be impaired by removing its last four C-terminal residues (Rsp5 Δ806). This mutant demonstrates a persistent Rsp5 HECT∼Ub thioester that can be consumed by β-ME, but not by lysine (Fig. S14A and B†), presumably because it cannot form a stable ligation conformation which is stabilized by the C-terminal –4Phe residue,^12^,^30^ or otherwise has reduced chemical reactivity to the incoming lysine acceptor.

In a UbFluor MT assay, Rsp5 Δ806 does not reach the activity of WT Rsp5 even at 100 mM lysine, while the Rsp5 E491A mutant does (Fig. 5A). This result is significant, because different reactivities of ligation defective HECT E3∼Ub thioesters to lysine were not previously appreciated. In addition to Rsp5 HECT E491A and Rsp5 HECT Δ806 mutants, Rsp5 HECT D495A and Rsp5 HECT E502A also demonstrate ligation-specific defects. These mutants form stable HECT∼Ub thioesters and demonstrate MT rates that increase in the presence of lysine although to a lesser extent than the E491A mutant, and ST rates that do not (Fig. S14E and S15†). Taken together, our results show that UbFluor can detect and quantitatively assess residues that control the chemical reactivity of HECT E3∼Ub thioesters toward lysine nucleophiles. Although the Rsp5 E491A, Rsp5 D495A, Rsp5 E502A, and Rsp5 Δ806 Ub thioesters have similar reactivities to β-ME, their reactivities toward lysine are distinct (Fig. S14E and S15†).

### 8. The UbFluor MT assay is a feasible HTS platform for a wide range of HECT E3 ligases.

Having established that the UbFluor MT assay can report the ligation and transthiolation defects of Rsp5 caused by mutations to the HECT domain, we were eager to explore the quantitative suitability of UbFluor for high-throughput screening using iodoacetamide as a small molecule HECT E3 inhibitor.

**Fig. 6:**
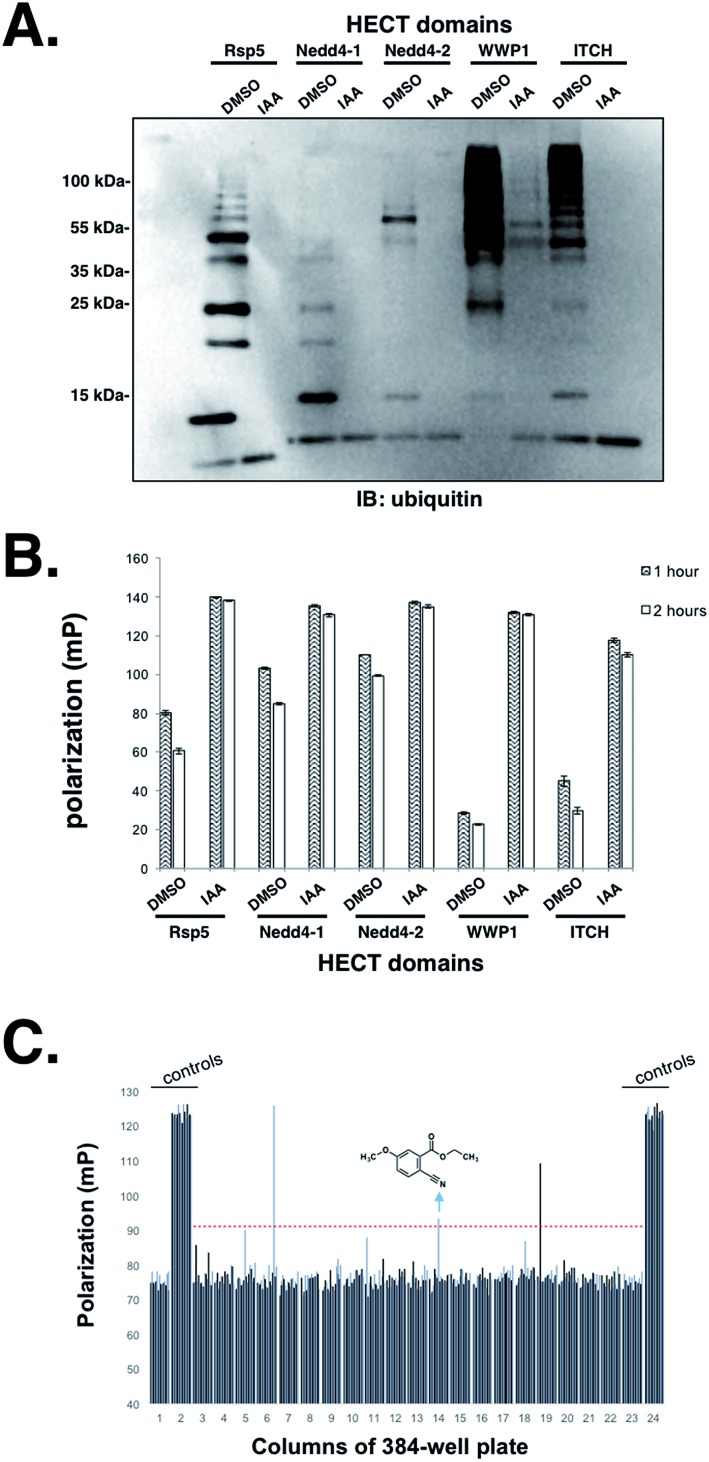
(A) Ligase (1.0 μM) was incubated in 50 mM HEPES pH 7.5, 50 mM NaCl, 0.5 mM TCEP, 6 μM Tween-20 with DMSO (0.2%) or DMSO with iodoacetamide (IAA, 1 mM) for 1 hour. UbFluor (5 μM) was then added and incubated with ligase for 90 minutes before quenching with reducing Laemmli buffer and resolving with SDS-PAGE. (B) Endpoint fluorescence polarization was measured for the same reactions of (A). Means ± SEM from 3 separate reaction trials are plotted. (C) Screening Maybridge small molecules against Nedd4-1 HECT. Endpoint fluorescence polarization assay in a 384-well plate where each bar represents the polarization measured in a given well. Nedd4-1 HECT (0.5 μM) was pre-incubated with small molecules (50 μM, 0.5% DMSO final concentration) for 1 hour in 50 mM HEPES pH 7.5, 150 mM NaCl, 6 μM Tween-20. UbFluor (5 μM final concentration) was then added and incubated with reaction solutions at 27 °C for 5 hours. Plate columns 1 and 23 were controls with 0.5% DMSO and columns 2 and 24 were controls with 1 mM iodoacetamide. The red line hit-threshold indicates 10 DMSO control standard deviations (10 × 1.60 mP) above the DMSO control average (75.6 mP).

Iodoacetamide alkylates the catalytic cysteine of HECT E3 and inactivates the enzyme. A *Z*′ score measures the suitability of a screening assay where assays that provide *Z*′ > 0.5 are considered robust.^32^ Gratifyingly, we observed a *Z*′ score of 0.72 when an Rsp5 human homologue Nedd4-1 (0.5 μM) was treated with 1 mM iodoacetamide (0.2% DMSO final concentration) *vs.* DMSO alone in the presence of 5 μM UbFluor in a 384-well plate (Fig. S16†). Further, we show the general utility of UbFluor for studying the biochemical mechanisms or for chemical probe discovery for other HECT ligases by treating UbFluor with HECT domains from the human ligases Nedd4-1, Nedd4-2, WWP1, and ITCH in the presence or absence of iodoacetamide (Fig. 6A and B and S17†). In all instances we observed real-time decay of the FP signal and the formation of poly-ubiquitin chains from UbFluor, suggesting that UbFluor is processed by these ligases *via* native-type mechanisms.

Encouraged by these control experiments, we screened the Nedd4-1 HECT domain against 320 small molecules from the Maybridge HitFinder Collection (Fig. 6C) and against 160 molecules from the MicroSource Spectrum Collection (Fig. S18†). The purpose of this experiment was not to settle upon an optimized inhibitor, but rather to show that a collection of small molecules can be screened against HECT E3s under our assay conditions. In these 384-well plate assays, Nedd4-1 HECT (0.5 μM) was pre-incubated with small molecules (50 μM, 0.5% DMSO final concentration) for 1 hour before adding UbFluor (5 μM final concentration). Fluorescence polarization was then measured every hour for 5 hours (ESI†). For controls, each plate contained 32 reactions with only DMSO (0.5%), and 32 reactions with iodoacetamide (1 mM). When screened compounds were plated in duplicate, similar polarization signals resulted for a given compound, thus further validating the robustness of the assay indicated by the strong *Z*′ score (Fig. S18†).

Setting the threshold for hit selection on the Maybridge plate to 10 standard deviations above the polarization of the DMSO controls, the hit rate is <1% (Fig. 6C, red dotted line). Following this primary screening experiment, we obtained a fresh solid sample of the arrow-indicated hit from Maybridge and observed it to inhibit substrate ubiquitination in a native ubiquitination reaction with E1 and E2 enzymes present (Fig. S19†). The two other hits were either an undesirable structure or had a solubility problem when purchased fresh.

This early validation of the screening assay indicates that UbFluor can be used to discover chemical probes for HECT ligases. It is important to note, however, that the mechanism of inhibition of the identified small molecule needs to be further investigated, and that it should not yet be considered an optimized Nedd4-1 probe. Furthermore, it contains 15 non-hydrogen atoms, which classifies it more as a fragment. Therefore, UbFluor can probably be used for high-content screening of fragments, as was done for the clinically approved drug vemurafenib.^33^

## Conclusion

In summary, we have introduced a novel fluorescent thioester probe UbFluor to quantify the enzymatic activity of HECT E3 ligases, which are genetically implicated in many human diseases.^16^,^34^–^37^ The unique feature of an UbFluor-based assay is its simplicity and quantitative real-time read-out: only two reagents are needed (UbFluor and HECT E3) to provide a homogeneous assay that can be run in a 384-well plate with continuous monitoring of the reaction progress. Therefore, UbFluor assays offer significant advantages over SDS-PAGE assays for kinetic studies, and have a wide dynamic range. Another advantage of UbFluor is its convenience for kinetic studies. The typical timescale of the native ubiquitination reaction is seconds, and necessitates quenching of the reaction mixture at sub-second intervals, often with a stopped-flow apparatus for kinetic studies.^12^ UbFluor reactions proceed on a minute timescale and can be continuously monitored without quenching. Remarkably, by simply changing the ratio of UbFluor : HECT E3, we can quantitatively describe defects in either transthiolation, or transthiolation + isopeptide ligation during HECT E3 catalysis. Using the ST condition, we conducted mechanistic investigations for E2-independent UbFluor transthiolation and discovered that it uses a similar, but distinct, mechanism to that of native E2∼Ub transthiolation: the Ub/C-lobe interface and catalytic loop of HECT E3 are essential for UbFluor transthiolation, but the Ub/N-lobe interface and E2 binding site are not. Measuring the rates of UbFluor consumption by HECT E3 under ST or MT conditions revealed that UbFluor can be used to identify inhibitory point mutations in HECT E3 transthiolation or isopeptide ligation in a manner that strongly correlates with the native ubiquitination reaction.

Furthermore, UbFluor allows the quantification of distinct chemical reactivities of HECT E3∼Ub thioesters that are defective in isopeptide ligation. We observed that both E491A and Δ806 Rsp5 mutants are defective in isopeptide ligation, and form the corresponding inactive Rsp5∼Ub thioesters. However, the E491A Rsp5∼Ub thioester is reactive toward lysine and its activity under MT conditions can be completely rescued with 100 mM lysine, while the Δ806 Rsp5∼Ub thioester is less reactive toward lysine such that its consumption of UbFluor under MT conditions can be rescued. These distinct chemical reactivities of isopeptide ligation defective mutant Rsp5∼Ub thioesters were not previously appreciated. Finally we have shown the utility of UbFluor in HTS assays by conducting the first proof of concept HTS.

Taken together, the development of UbFluor outlined here opens the path forward to discover chemical probes for HECT E3s and perhaps other cysteine containing E3s such as RBR E3s, bacterial HECT-like E3s, and NELs (∼70 enzymes) to explore their biological functions and validate these enzymes as novel classes of drug targets.^38^–^41^ However, due to the complex regulatory mechanisms of these E3s that include intramolecular auto-inhibitory interactions, post-translational modifications, allosteric activation by phosphorylated ubiquitin and substrates, separate studies are needed to validate the utility of UbFluor for bacterial HECT-like E3s, NEL, and RBR E3s.

## Supplementary Material

Supplementary information
