# Supplementary material for: UbFluor: a mechanism-based probe for HECT E3 ligases
Source: Chem Sci. 2016 May 17;7(8):5587–95. doi: 10.1039/c6sc01167e (PMC4965700; doi:10.1039/c6sc01167e)
Supplement: Supplementary file 1 [file SC-007-C6SC01167E-s001.pdf]

Electronic Supplementary Material (ESI) for Chemical Science.
This journal is © The Royal Society of Chemistry 2016

Supporting Information

UbFluor: A Mechanism-Based Probe for HECT E3 Ligases

David T. Krist,[1] Sungjin Park,[1,2] Galyah H. Boneh,[1] Sarah E. Rice[2] and Alexander V. Statsyuk[1*]

[1]*Department of Chemistry, Chemistry of Life Processes Institute, Northwestern University, Silverman Hall, 2145 Sheridan Road, Evanston, Illinois 60208, USA*

[2]*Department of Cell and Molecular Biology, Feinberg School of Medicine, Northwestern University, 303 East Chicago Avenue, Chicago, Illinois, 60611, USA*

Table of Contents

Supporting Data **S2**

Supplementary Methods **S28**

Biochemical Procedures **S28**

Protein Purification **S36**

Supplemental References **S41**

Supporting Data

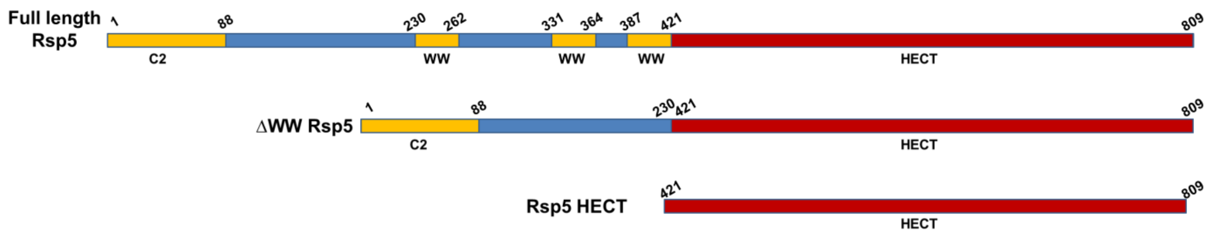

Figure S1. Domain map of Rsp5. We used the ΔWW Rsp5 construct and Rsp5 HECT construct in this study. The ΔWW Rsp5 construct used in this work and in our previous study was found to harbour the mutations N463D and F512S (numbered according to full length Rsp5).[1] To the best of our knowledge, these mutations do not affect the catalytic mechanism. These mutations were corrected during cloning of the HECT domain construct.

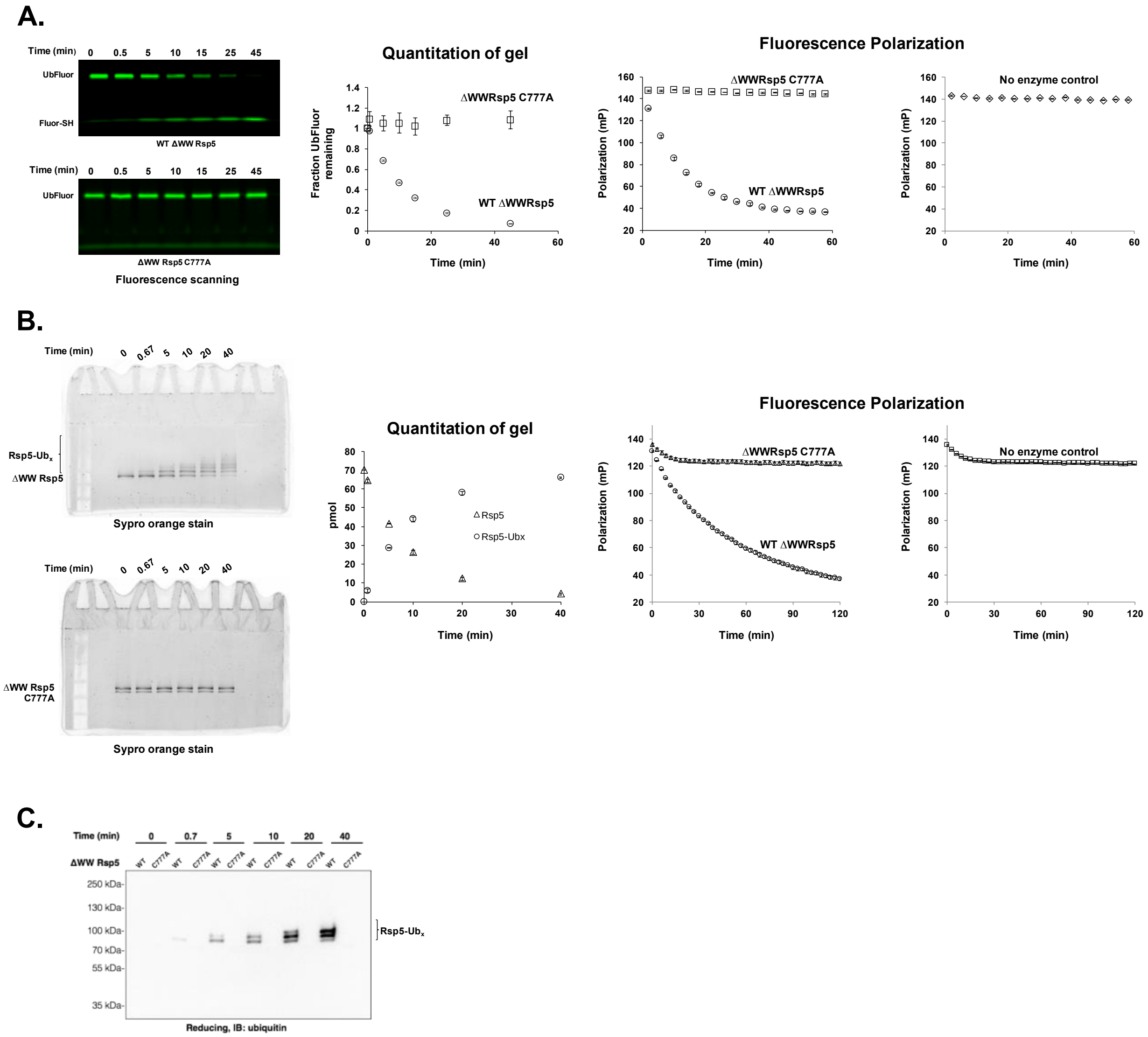

Figure S2. Initial validation of UbFluor (A) Single turnover conditions were used: UbFluor (1.0 µM) was incubated with ΔWW Rsp5 (5.0 µM, circles), the catalytically inactive ΔWW Rsp5 C777A (5.0 µM, squares), or no enzyme (diamonds) in 150 mM NaCl, 6 µM Tween-20, 0.5 mM TCEP, 50 mM HEPES pH 7.5. Reactions were analysed at the indicated time points with gel or fluorescence polarization in a 384-well plate with the Synergy4 plate reader. The gels and fluorescence polarization graph are also shown in Figure 2 of the main text. The graphs show mean ± SEM from three separate trials. (B) Multiturnover conditions were used: UbFluor (5.0 µM) was incubated with ΔWW Rsp5 (1.0 µM, circles), the catalytically inactive ΔWW Rsp5 C777A (1.0 µM, triangles), or no enzyme (squares) in 150 mM NaCl, 6 µM Tween-20, 0.5 mM TCEP, 50 mM HEPES pH 7.5. Reactions were analysed at the indicated time points with gel or fluorescence polarization in a 384-well plate with the Synergy4 plate reader. The graphs show

mean ± SEM from three separate trials. Auto-ubiquitinated ΔWW Rsp5 is marked at Rsp5-Ub_x. (C) The reactions of (B) were analysed with α-ubiquitin western blot to show that the Rsp5 catalytic cysteine Cys^{777} is required to process UbFluor.

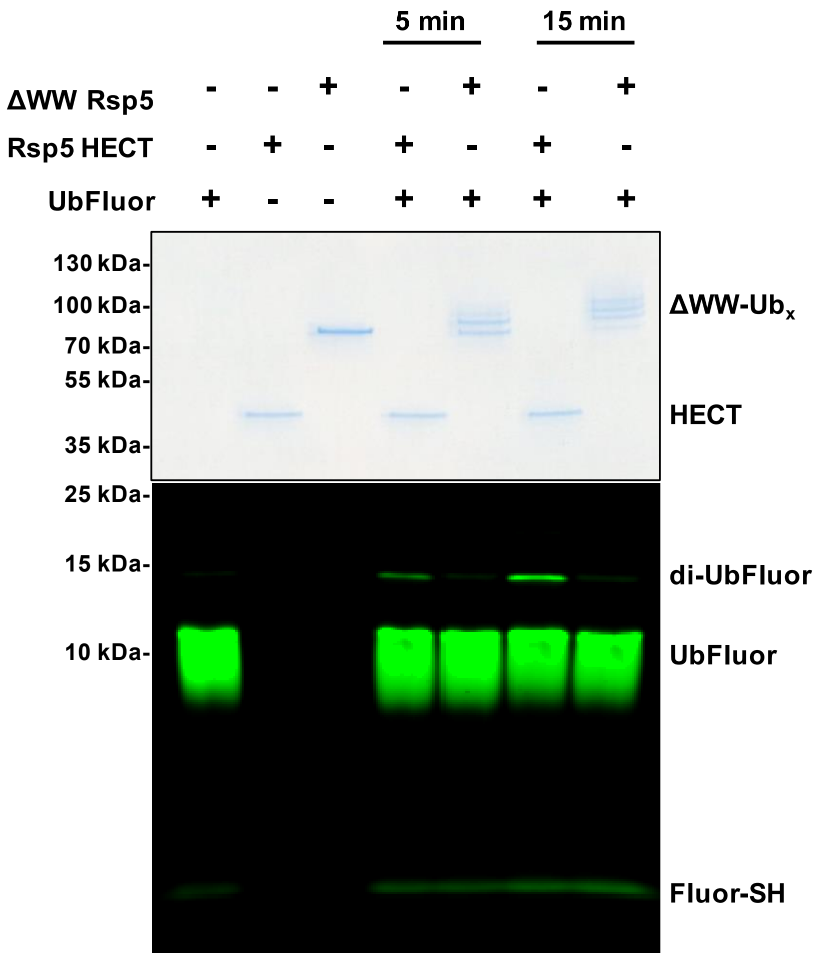

Figure S3. The ligation product of a Rsp5/UbFluor reaction with ΔWW Rsp5 is auto-ubiquitinated ligase. In a reaction with the Rsp5 HECT domain, the product is di-UbFluor. UbFluor (10 µM) was incubated with the indicated construct of Rsp5 (1 µM) in 50 mM HEPES 7.5, 150 mM NaCl, 6 µM Tween-20, 0.5 mM TCEP at 25 °C for the indicated time. Reactions were quenched with non-reducing Laemmli buffer. Gels were imaged by Coomassie stain (top) or fluorescence scanning (bottom).

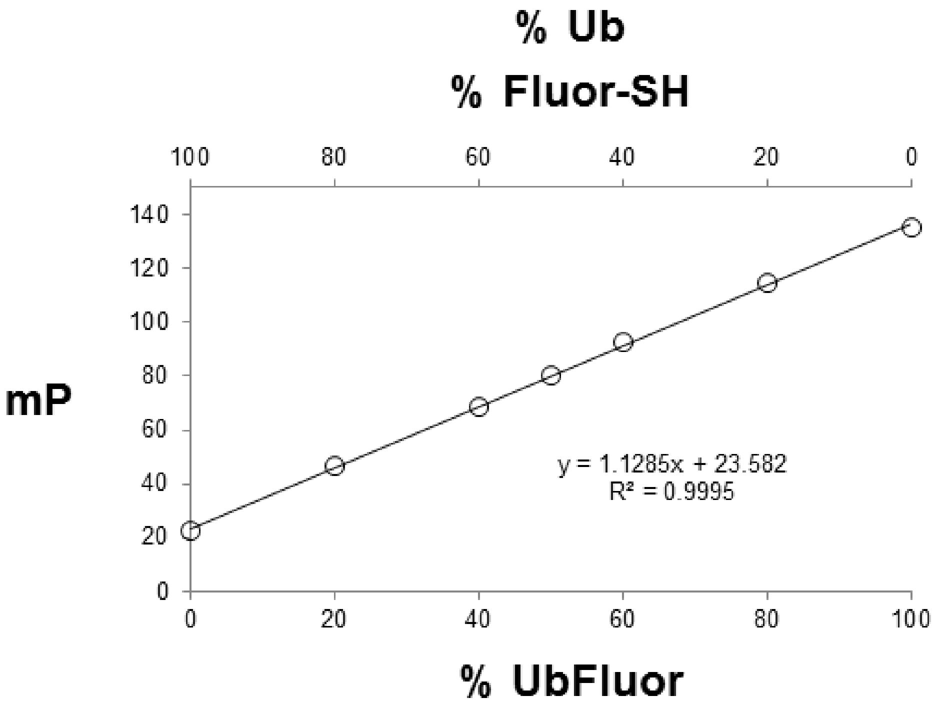

Figure S4. There is a linear relationship between UbFluor concentration and polarization. Mixtures of UbFluor (0 – 5 μM), Ub (0 – 5 μM), and Fluor-SH (0 – 5 μM) were incubated in 150 mM NaCl, 50 mM HEPES pH 7.5, 6 μM Tween-20, and 0.5 mM TCEP. The solutions were loaded to wells in a 384 well low volume, low binding plate (Corning 3820). Fluorescence polarization for each solution was read on the Synergy4 plate reader.

**Single turnover**	**Conversion**
0.25 μM UbFluor	[UbFluor] = 0.002[mP] - 0.06
0.50 μM UbFluor	[UbFluor] = 0.004[mP] - 0.12
0.75 μM UbFluor	[UbFluor] = 0.006[mP] - 0.18
1.00 μM UbFluor	[UbFluor] = 0.008[mP] - 0.24
**Multiturnover**	**Conversion**
10 μM UbFluor	[UbFluor] = 0.09259[mP] - 2.77778
12.5 μM UbFluor	[UbFluor] = 0.11574[mP] - 3.47222
15 μM UbFluor	[UbFluor] = 0.13889[mP] - 4.16667
20 μM UbFluor	[UbFluor] = 0.18519[mP] - 5.55556

Table S1. To convert polarization to units of UbFluor concentration, linear transformations were calculated according to observations taken at the beginning of our study that polarization at maximal UbFluor concentrations (100% UbFluor) = ~155 mP under single turnover conditions (0.25 – 1.00 μM UbFluor), and that polarization at maximal UbFluor concentrations (100% UbFluor) = ~138 mP under multiturnover conditions (10 – 20 μM UbFluor). Under single and multiturnover conditions, the minimal polarization after complete UbFluor conversion = ~30 mP (0% UbFluor). Over the course of our study to compare Rsp5 mutants (Figure 4), the initial polarization values of 155 mP and 138 mP did not vary by more than 2-3 mP. Between different batches of prepared UbFluor, the initial polarization values did not vary by more than 15 mP; in these cases the linear transformations were adjusted accordingly. Additionally, care was maintained to use a single UbFluor batch for all relevant comparisons within a given data set.

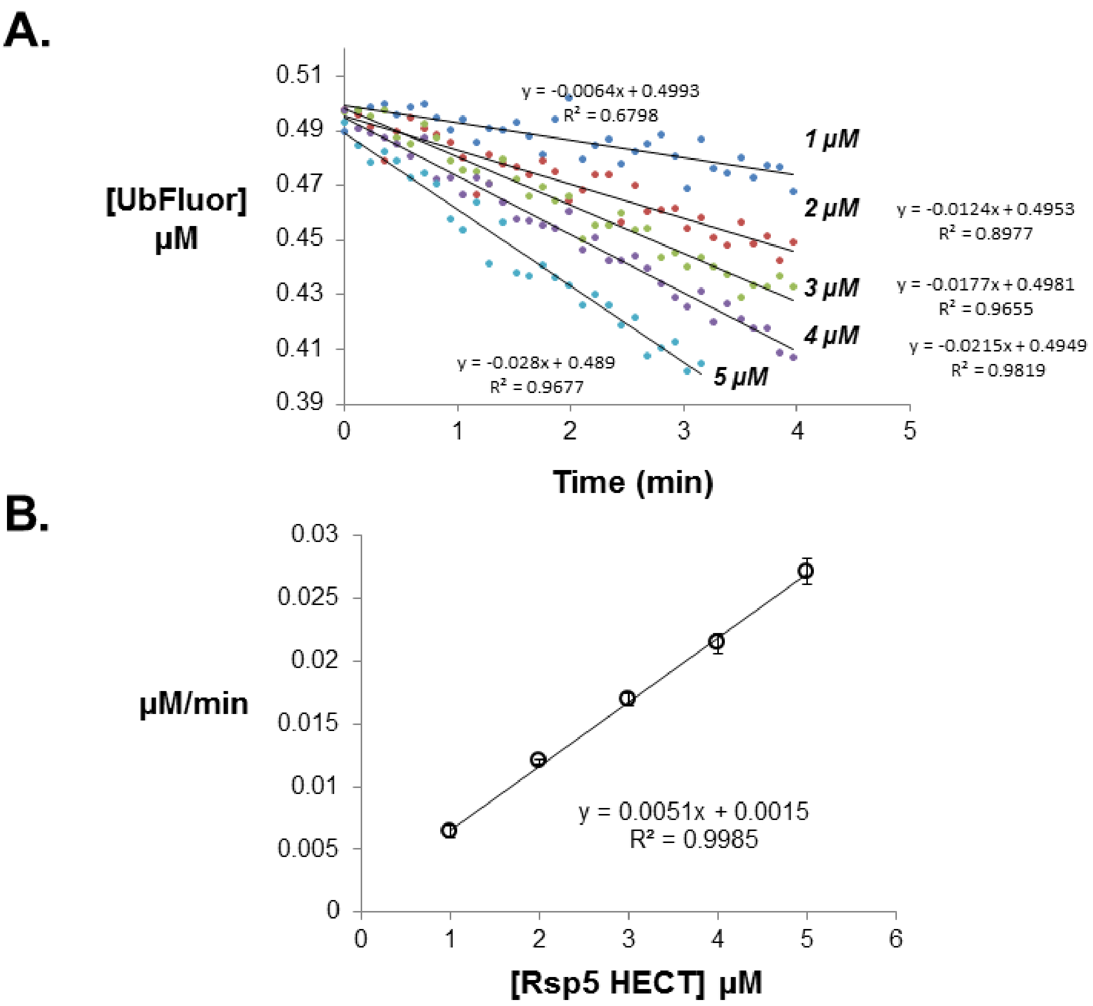

Figure S5. The consumption of UbFluor proceeds linearly with enzyme concentration (A) UbFluor (0.5 μM) was incubated with the indicated concentrations of WT Rsp5 HECT (1 – 5 μM) in 50 mM HEPES 7.5, 150 mM NaCl, 6 μM Tween-20, 0.5 mM TCEP at 25 °C. Reactions were monitored for 4 minutes, taking FP readings every 7 seconds. FP data was converted to UbFluor concentration according to Table S1. Data after 3 minutes with 5 μM Rsp5 HECT was not used since more than 20% of the UbFluor was consumed past this time. (B) Average rates for each concentration of Rsp5 HECT are plotted. n =3. Error: +/- SEM.

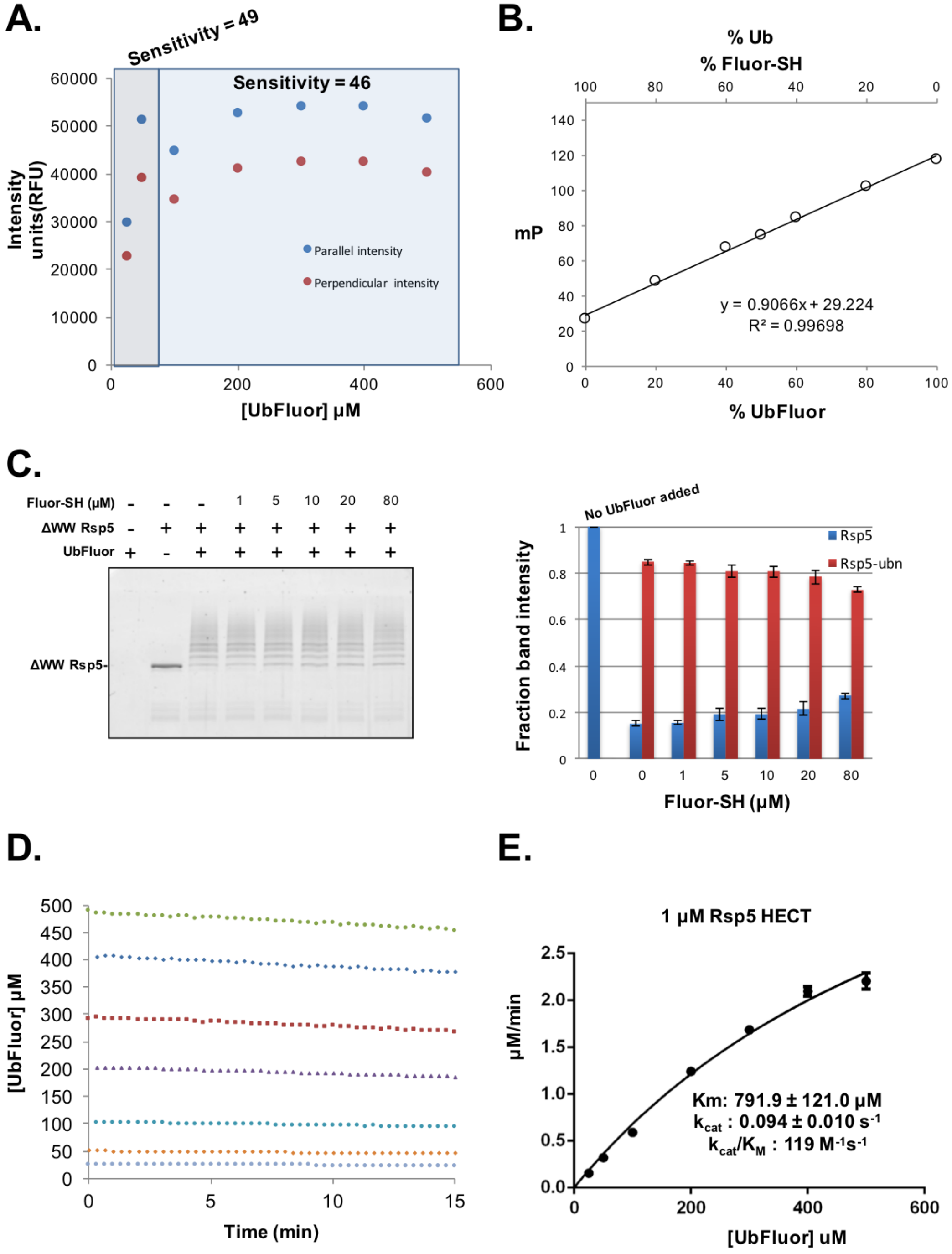

Figure S6. The Rsp5-UbFluor reaction can be modelled by Michaelis-Menten kinetics. (A) Reactions contained WT Rsp5 HECT (1 μM) and UbFluor in 50 mM HEPES 7.5, 150 mM NaCl, 6 μM Tween-20, 0.5 mM TCEP at 25 °C. Reactions were monitored for 20 minutes, taking FP measurements every 20 seconds. The averaged fluorescence intensities are plotted

from these experiments. Sensitivity is a setting on the Synergy4 plate reader that adjusts the detection of fluorescence intensity. (B) Mixtures of UbFluor, Ub, Fluor-SH, and Rsp5 HECT C777A (1 μM) were incubated in 150 mM NaCl, 50 mM HEPES pH 7.5, 6 μM Tween-20, and 0.5 mM TCEP. The total fluorescein in each mixture as either Fluor-SH or UbFluor was 400 μM. The solutions were loaded to wells in a 384 well low volume, low binding plate (Corning 3820). Fluorescence polarization was read on the Synergy4 plate reader over 2 minutes and averaged. Sensitivity was set to 46. (C) ΔWW Rsp5 (1 μM) was incubated with UbFluor (10 μM) and the indicated amount of free Fluor-SH in 50 mM NaCl, 20 mM HEPES pH 7.2 for 1 hour at room temperature. Reactions were quenched with Laemmli buffer and 7.8 μM β-ME. Products were separated on a 10% SDS-PAGE gel and analysed by Sypro Orange staining. Quantitation of the gel bands: the bar graph shows the intensity of unmodified Rsp5 normalized against the sum of all band intensities in a given lane. The same is done for ubiquitinated Rsp5 The bar graph plots mean ± SEM from 3 separate trials on 3 separate gels. Bands were quantified with ImageJ. (D) Fluorescence polarization data from (A) is plotted. To produce this plot, crude polarization values were subtracted against a 'no enzyme' control with the same UbFluor concentration. (E) Data from (D) was fit with GraphPad Prism software (Michaelis Menten non-linear regression). Triplicate experiments were performed at each concentration to plot mean ± SEM.

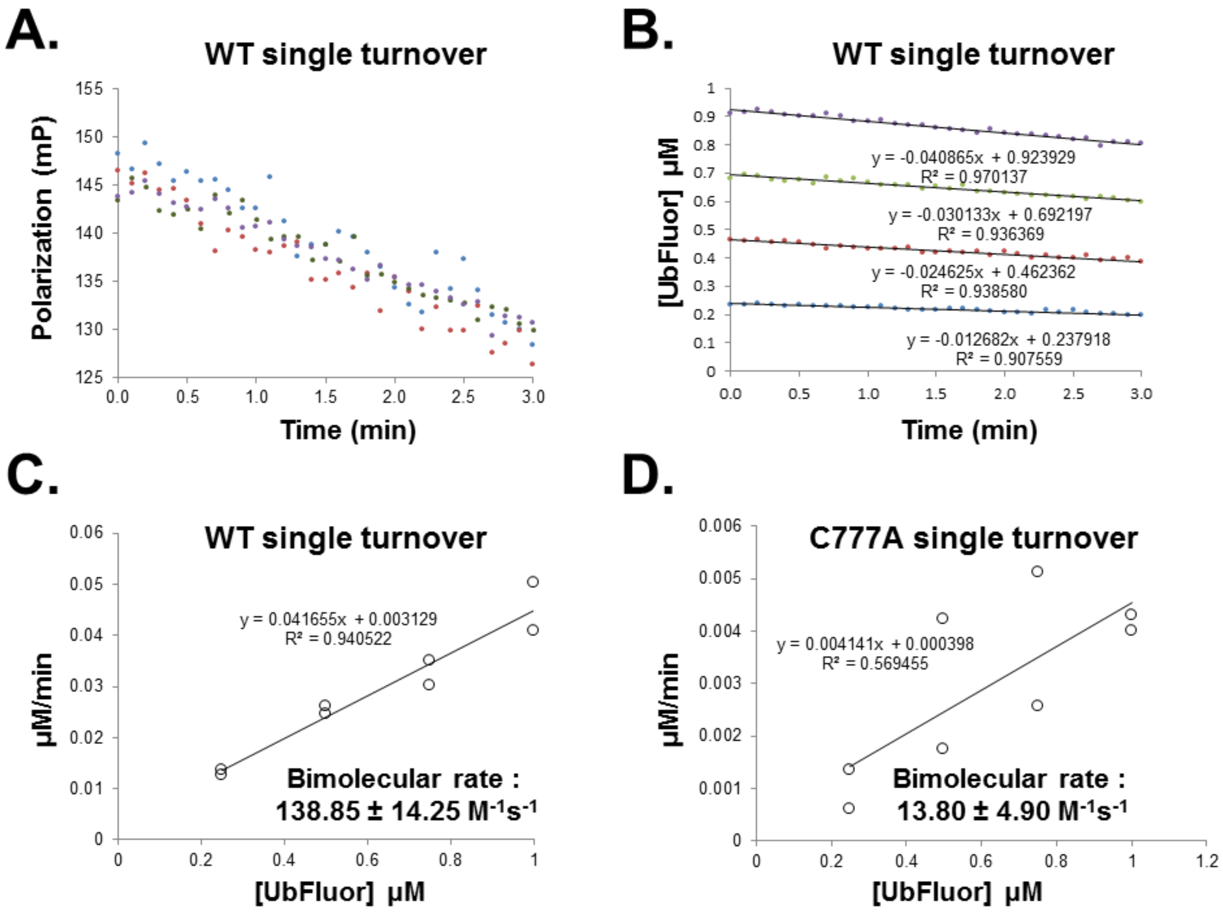

Figure S7. A bimolecular rate constant can be obtained from single turnover conditions. (A) Raw polarization data from a single turnover experiment with WT Rsp5 HECT: Rsp5 HECT (5 µM) with UbFluor (0.25 (blue), 0.50 (red), 0.75 (green), 1.00 µM (purple)). Measurements were taken every 6 seconds for 3 minutes, at which point 20% of available UbFluor had been consumed. (B) Polarization values from (A) are converted to concentrations of UbFluor according to Table S1. (C) Slopes from the lines in (B) and from a replicate experiment performed at the same enzyme and UbFluor concentrations are plotted against UbFluor concentrations. The resulting slope of the line (k_{obs} = 0.041655 min^{-1}) is converted into the bimolecular rate by dividing by enzyme concentration and 60 sec/min. (D) The same procedure described for (A – C) is performed for the catalytically inactive mutant Rsp5 HECT C777A. For plots in (C) and (D), error of bimolecular rate was calculated using the “linest” function in Microsoft Excel to specify the error in the slope. The R^2 values for linear trendlines in panel A

are > 0.90. Data in panel A appear more scattered than in panel B because of the y-axis scale difference. Measurement of UbFluor consumption at each of four UbFluor concentrations was performed twice (8 total measurements). All data points are shown in (C) and (D).

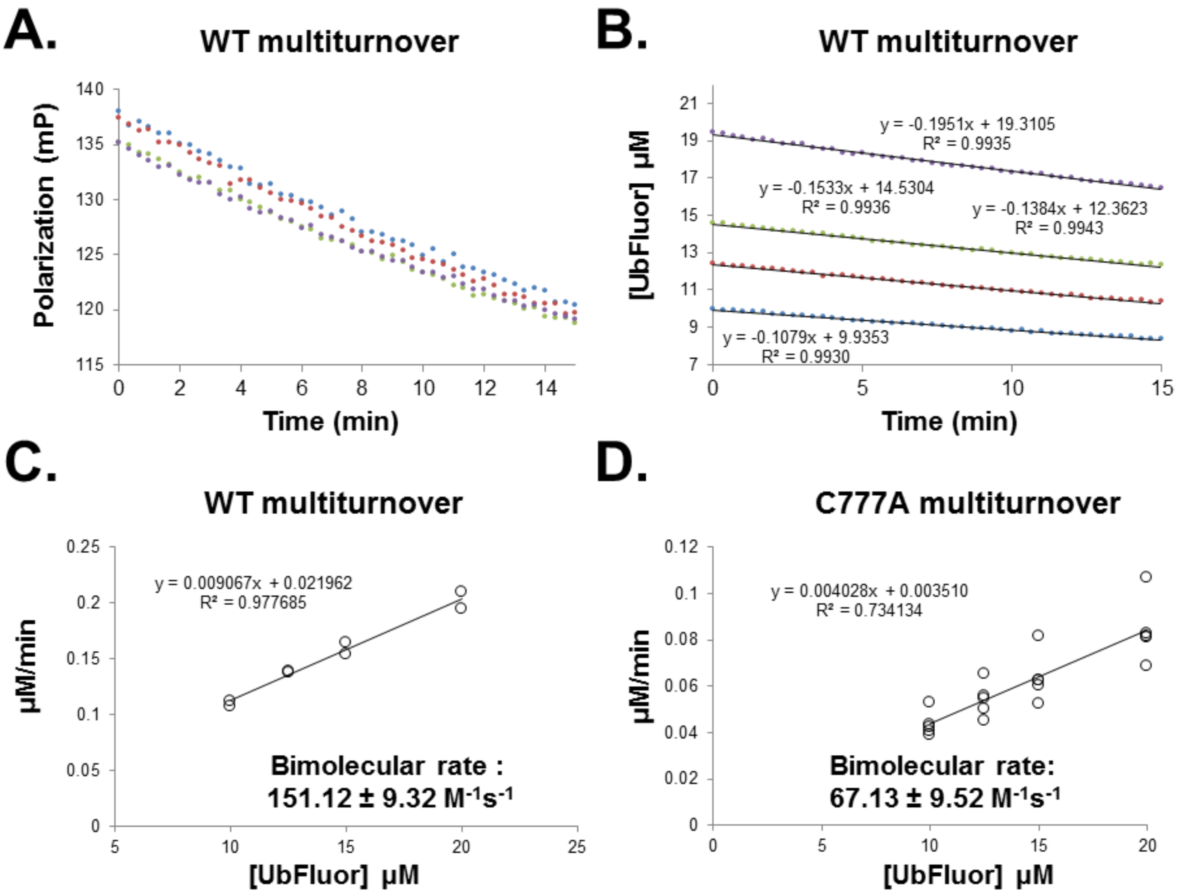

Figure S8. A bimolecular rate constant can be obtained from multiturnover conditions. (A – C are also given as main text Figure 3). (A) Raw polarization data from a multiturnover experiment with WT Rsp5 HECT: Rsp5 HECT (1 µM) with UbFluor (10 (blue), 12.5 (red), 15 (green), 20 µM (purple)). Measurements were taken every 20 seconds for 15 minutes. (B) Polarization values from (A) are converted to concentrations of UbFluor according to Table S1. (C) Slopes from the lines in (B) and from a replicate experiment performed at the same enzyme and UbFluor concentrations are plotted against UbFluor concentrations. The resulting slope of the line (k_{obs} = 0.009067 min^{-1}) is converted into the bimolecular rate by dividing by enzyme concentration and 60 sec/min. (D) The same procedure described for (A – C) is performed for the catalytically inactive mutant Rsp5 HECT C777A. For plots in (C) and (D), error of bimolecular rate was calculated using the "linest" function in Microsoft Excel to specify the error in the slope. Measurement of UbFluor consumption at each of four UbFluor concentrations was performed twice for WT (8 total measurements), and was performed 5 times for C777A (20 total measurements). All data points are shown in (C) and (D).

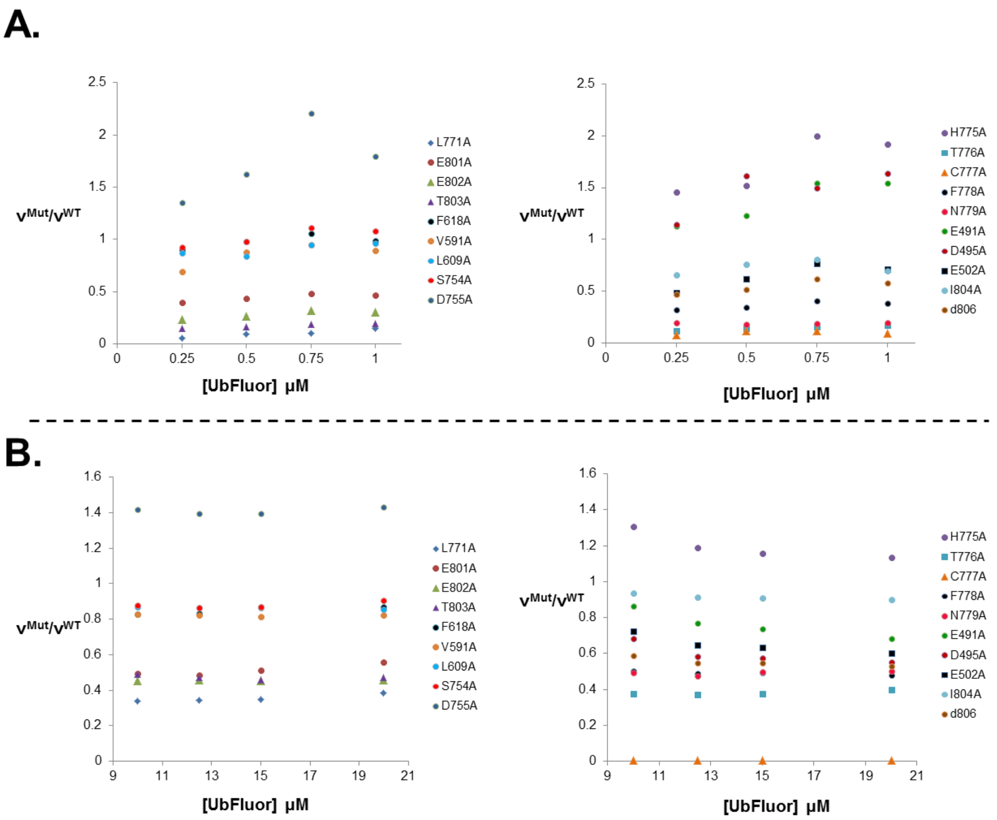

Figure S9. V^{mutant}/V^{WT} **shows that the ratio of mutant Rsp5 HECT to wild type Rsp5 HECT velocities is independent of UbFluor concentration.** (A) For single turnover experiments. (B) For multiturnover experiments.

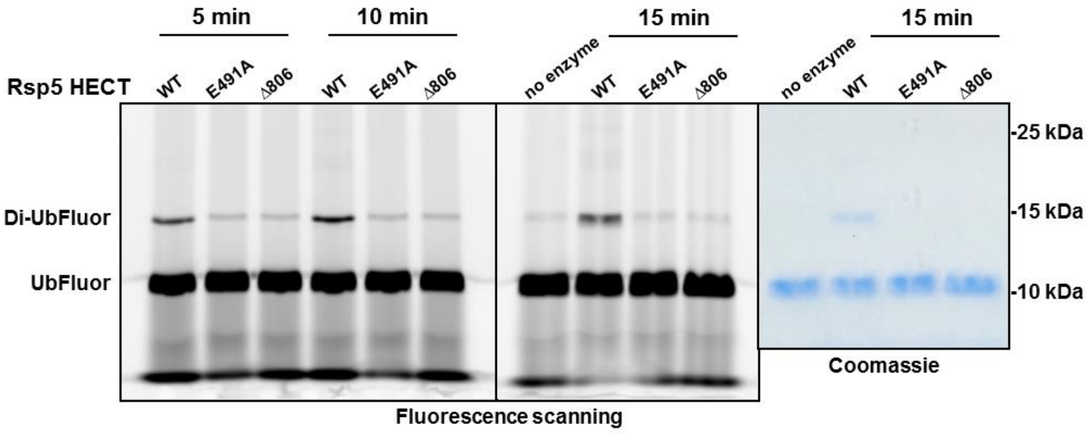

Figure S10. WT Rsp5 HECT produces Ub-UbFluor (di-UbFluor), while Rsp5 HECT E491A and Rsp5 Δ806 do not. Reactions with the indicated mutant of Rsp5 HECT (1 μM) and UbFluor (10 μM) in 50 mM HEPES 7.5, 150 mM NaCl, 6 μM Tween-20, 0.5 mM TCEP were incubated for the indicated amount of time and then quenched with non-reducing Laemmli buffer. Reaction solutions were resolved by SDS-PAGE and then imaged by fluorescence scanning or Coomassie stain.

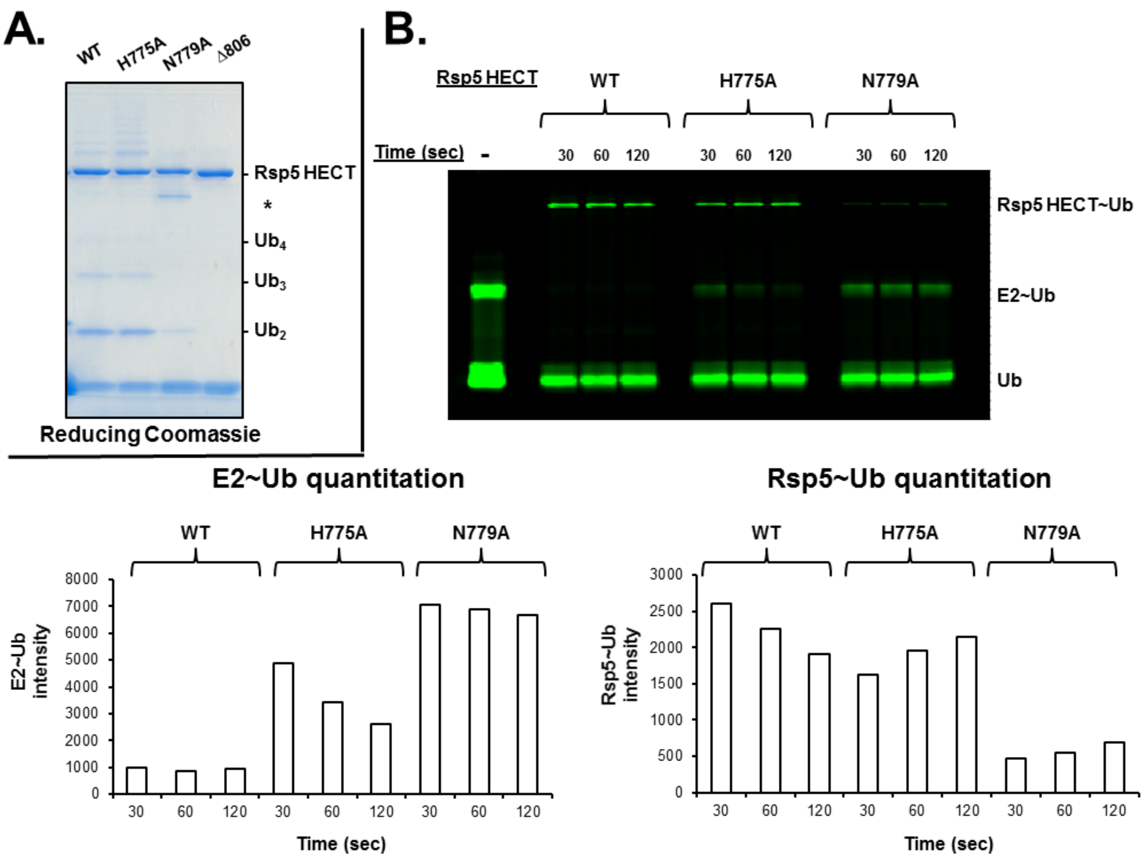

Figure S11. Rsp5 HECT H775A exhibits a defect in the native UbcH5B~Ub to HECT transthiolation but not in transthiolation with UbFluor. (A) Rsp5 HECT WT and Rsp5 HECT H775A produce an equivalent amount of di-ubiquitin, while Rsp5 HECT N779A is deficient. Rsp5 HECT (2 μM) was incubated with UbFluor (10 μM) in 25 mM HEPES 7.6, 100 mM NaCl, 4 mM $MgCl_2$ for 30 minutes at 23 °C and then quenched with 6X Laemmli buffer with β-ME. (B) Pulse-chase assay: Uba1 (E1, 0.42 μM), UbcH5B (E2, 9.1 μM), and fluorescent ubiquitin (16.7 μM) were incubated with 2 mM ATP, 10 mM $MgCl_2$, 300 mM NaCl, and 50 mM Tris pH 7.6 for 30 minutes at room temperature. The reaction was then diluted four-fold with 25 mM EDTA, 100 mM NaCl, 25 mM HEPES pH 7.5, and incubated on ice for 10 minutes to halt generation of UbcH5B~Ub. Each chase reaction was then run with ~0.4 μM UbcH5B~Ub, 2 μM Rsp5 in 300 mM NaCl, and 50 mM Tris pH 7.6 on ice, and quenched at the indicated time with non-reducing Laemmli buffer. The gel was imaged with fluorescent scanning (Typhoon 9400). * indicates a protein impurity from preparation of the N779A mutant. Note: in (B), fluorescent ubiquitin is not UbFluor.

Nedd4 family HECT ligases + E6AP aligned with uniprot.org

|RSP5_YEAST	VQQLPKSHTCFNRVDLPQYVDYD	790
|NEDD4-1_HUMAN	PEKLPRAHTCFNRLDLPPYESFE	1299
|NEDD4-2_HUMAN	PEKLPRAHTCFNRLDLPPYETFE	834
|SMURF1_HUMAN	TDNLPKAHTCFNRIDIPPYESYE	738
|SMURF2_HUMAN	TNNLPKAHTCFNRIDIPPYESYE	729
|WWP1_HUMAN	DTWLPRSHTCFNRLDLPPYKSYE	903
|WWP2_HUMAN	ETWLPRSHTCFNRLDLPPYKSYE	851
|ITCH_HUMAN	ENWLPRSHTCFNRLDLPPYKSYE	884
|NEDL1_HUMAN	ITSLPRAHTCFNRLDLPPYPSYS	1587
|NEDL2_HUMAN	ITALPRAHTCFNRLDLPPYPSFS	1553
|E6AP_HUMAN	TERLPTSHTCFNVLLLPEYSSKE	856

Table S2. Alignment of human Nedd4 family HECT ligases sequence near the catalytic cysteine. The conserved Rsp5 His^{775} is highlighted in cyan, the conserved catalytic cysteine Rsp5 Cys^{777} is highlighted in yellow, and the conserved Rsp5 Asn^{779} is highlighted in green.

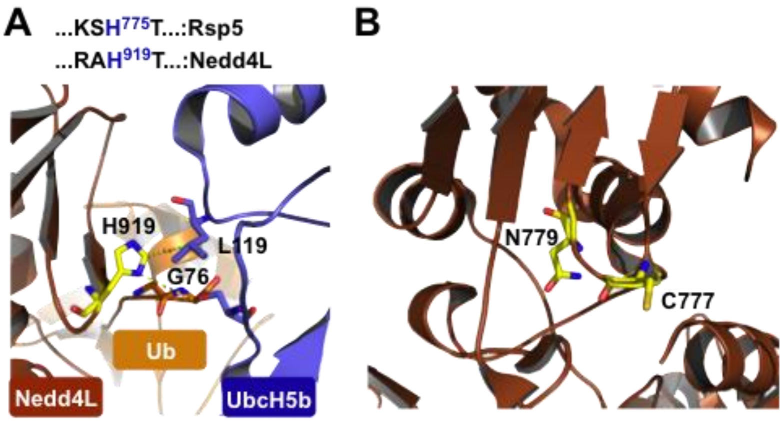

Figure S12. Previously published structures. (A) Co-crystal structure of Nedd4L HECT E3 bound to UbcH5B~Ub oxyester showing how H919, which is equivalent to H775 of Rsp5, anchors G76 of Ub and L119 of E2 enzyme to the active site of Nedd4L. PDB ID: 3JW0.[2] (B) N779 is shown in an Rsp5 crystal structure. PDB ID: 3OLM.[3] The involvement of N779 in the E2~Ub transthiolation reaction was not known before our study.

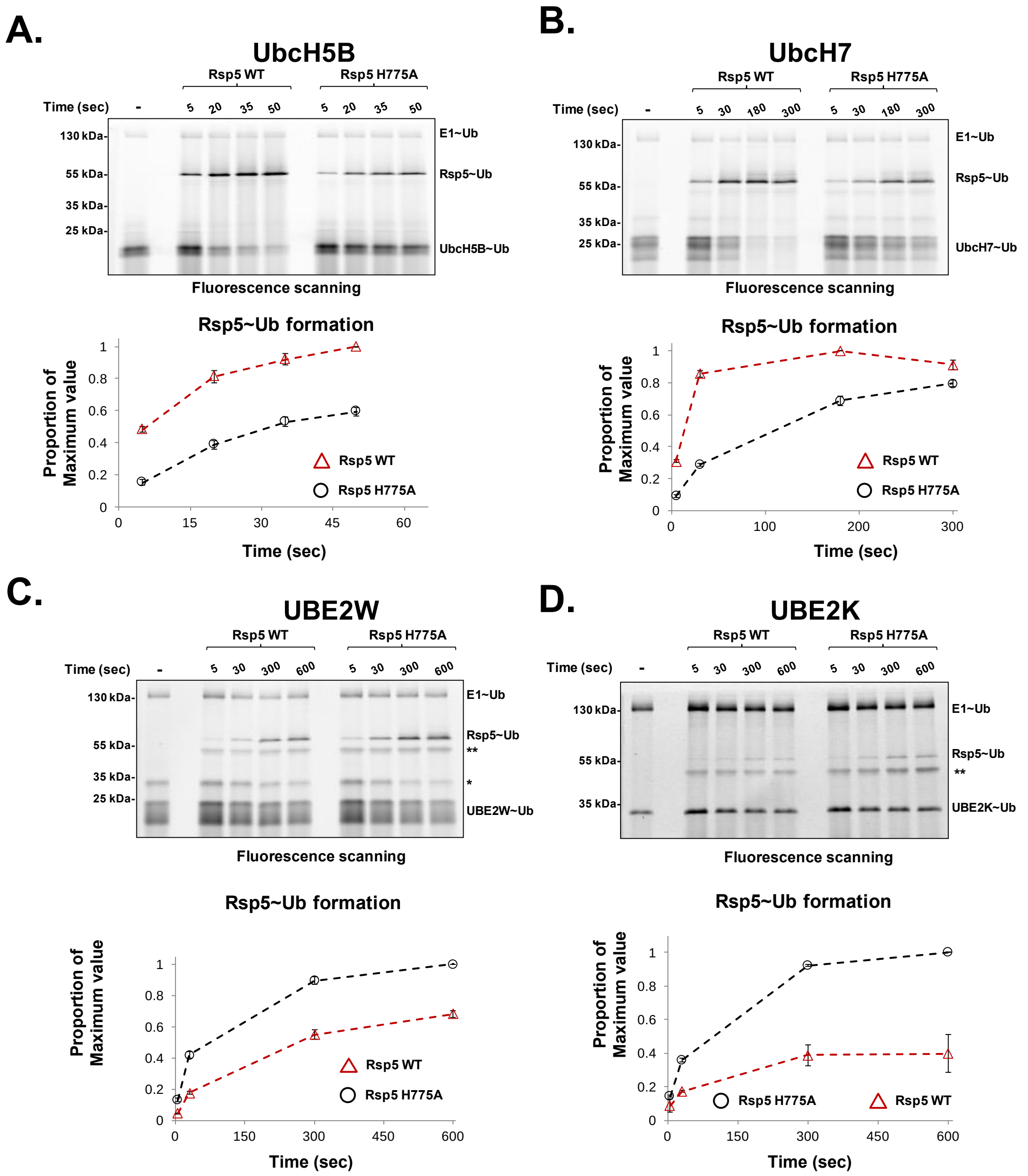

Figure S13. Rsp5 H775A reacts more readily with non-cognate E2~Ub (UBE2W and UBE2K) than with cognate E2~Ub (UbcH5B and UbcH7). (A) Pulse-chase assay to observe transfer of ubiquitin from UbcH5B~Ub to Rsp5 HECT WT or H775A. Pulse reactions with Uba1 (0.42 μM), UbcH5B (9.1 μM), and fluorescent ubiquitin (16.7 μM) were incubated with 2 mM ATP, 8 mM $MgCl_2$, 300 mM NaCl, and 50 mM Tris pH 7.6 for 30 minutes at room temperature. The reaction was then diluted four-fold with 25 mM EDTA, 100 mM NaCl, 25 mM HEPES pH 7.5, and then desalted into the same buffer to halt generation of E2~Ub. Each chase reaction was

then run with UbcH5B~Ub (~0.4 μM), Rsp5 (2 μM), in 300 mM NaCl, 50 mM Tris pH 7.6 on ice, and quenched at the indicated time with non-reducing Laemmli buffer. (B) With UbcH7, chase performed at room temperature. (C) With UBE2W, chase performed at room temperature. (D) With UBE2K, chase performed at room temperature. ** indicates a fluorescent Ub adduct that was built independent of the Rsp5 HECT catalytic cysteine. * indicates UBE2W~Ub-Ub. Cognate and non-cognate E2 enzymes were selected according to Sheng, et al.[4] Graphs in (A-D) have plotted mean ± SEM from 3 separate reaction trials run on separate gels. Note: the fluorescent ubiquitin used in these assays is not UbFluor.

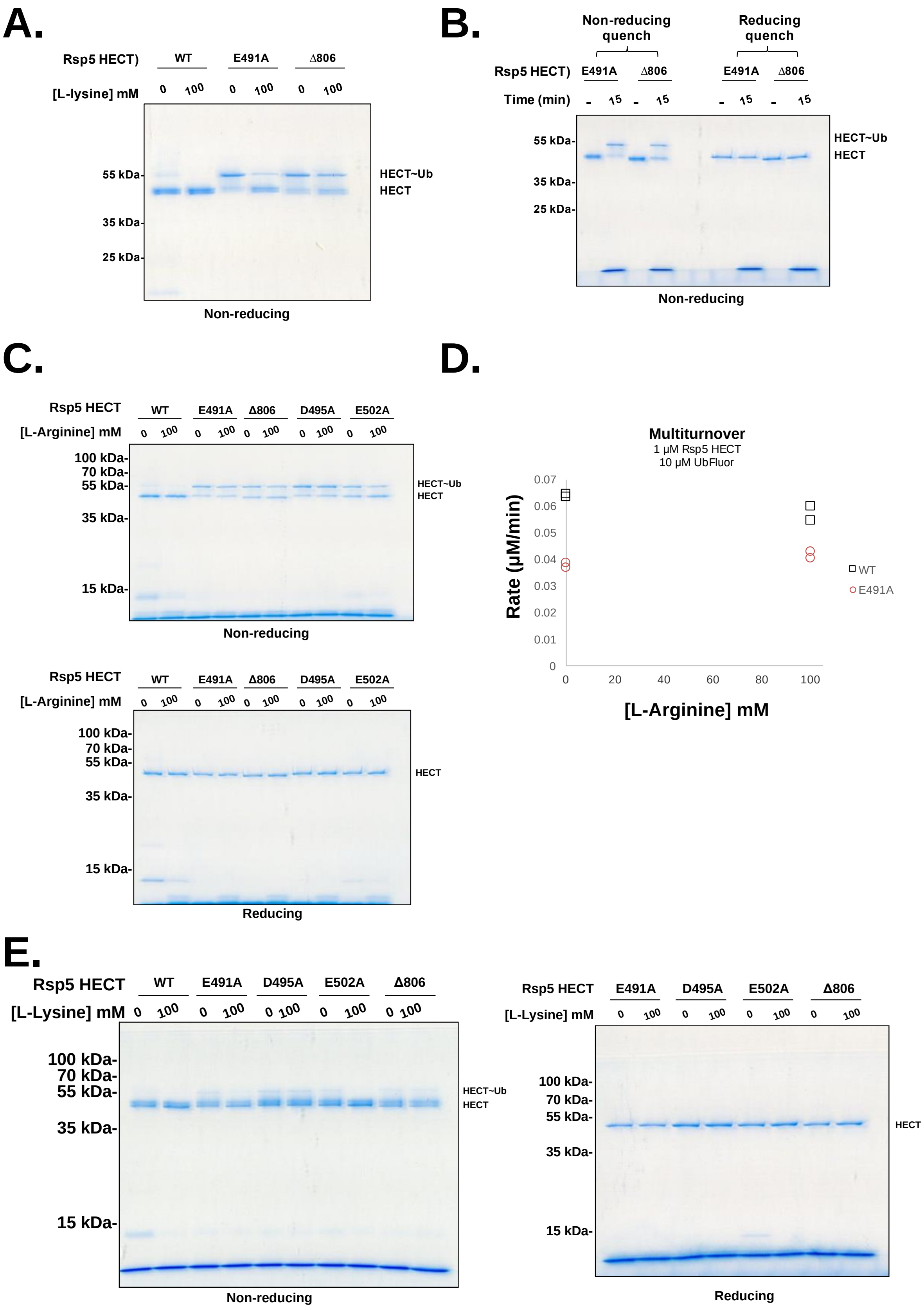
A.
Rsp5 HECT)
WT
E491A
Δ806
[L-lysine] mM
0
100
55 kDa-
35 kDa-
25 kDa-
HECT~Ub
HECT
Non-reducing
B.
Non-reducing quench
Reducing quench
Rsp5 HECT)
E491A
Δ806
Time (min)
-
15
55 kDa-
35 kDa-
25 kDa-
HECT~Ub
HECT
Non-reducing
C.
Rsp5 HECT
WT
E491A
Δ806
D495A
E502A
[L-Arginine] mM
0
100
100 kDa-
70 kDa-
55 kDa-
35 kDa-
15 kDa-
HECT~Ub
HECT
Non-reducing
Rsp5 HECT
WT
E491A
Δ806
D495A
E502A
[L-Arginine] mM
100 kDa-
70 kDa-
55 kDa-
35 kDa-
15 kDa-
HECT
Reducing
D.
Multiturnover
1 μM Rsp5 HECT
10 μM UbFluor
Rate (μM/min)
0.07
0.06
0.05
0.04
0.03
0.02
0.01
0
0
20
40
60
80
100
[L-Arginine] mM
WT
E491A
E.
Rsp5 HECT
WT
E491A
D495A
E502A
Δ806
[L-Lysine] mM
0
100
100 kDa-
70 kDa-
55 kDa-
35 kDa-
15 kDa-
HECT~Ub
HECT
Non-reducing
Rsp5 HECT
E491A
D495A
E502A
Δ806
[L-Lysine] mM
100 kDa-
70 kDa-
55 kDa-
35 kDa-
15 kDa-
HECT
Reducing

Figure S14. Rsp5 HECT~Ub thioesters can be consumed by L-lysine or β-ME but not by L-arginine. (A) Reactions with the indicated mutant of Rsp5 HECT (1 μM), UbFluor (10 μM) in 50 mM HEPES 7.5, 150 mM NaCl, 6 μM Tween-20, 0.5 mM TCEP, -/+ 100 mM L-lysine proceeded for 45 minutes at room temperature before quenching with non-reducing Laemmli buffer. (B) Reactions with the indicated mutant of Rsp5 HECT (1 μM), -/+ UbFluor (10 μM) in 50 mM HEPES 7.5, 150 mM NaCl, 6 μM Tween-20, 0.5 mM TCEP proceeded for the indicated time before quenching with non-reducing Laemmli buffer or Laemmli buffer with β-mercaptoethanol (1.2 M). Reduced samples were further incubated at 95 °C before loading to the gel. (C) Same conditions as (A) but with L-arginine rather than L-lysine. (D) MT initial velocities were measured with fluorescence polarization. Reactions were run in 150 mM NaCl, 0.5 mM TCEP, 6 μM Tween-20, 50 mM HEPES pH 7.5 with the indicated amount of L-arginine. The indicated amount of L-arginine was added as part of a 10X buffer (pH was then adjusted to 7.5 for each 10X buffer). A background subtraction of raw polarization data was performed based on reactions without ligase at each concentration of L-arginine. Two replicates were performed for each ligase and concentration, all data is shown. (E) 15 minute reactions: the same conditions as in (A) were used to analyse Rsp5 HECT mutants. However, reactions were only run for 15 minutes before quenching. 15 minutes is a relevant timeframe since this was the duration of the MT FP assays. Gels for Figure S14 were visualized with Coomassie stain

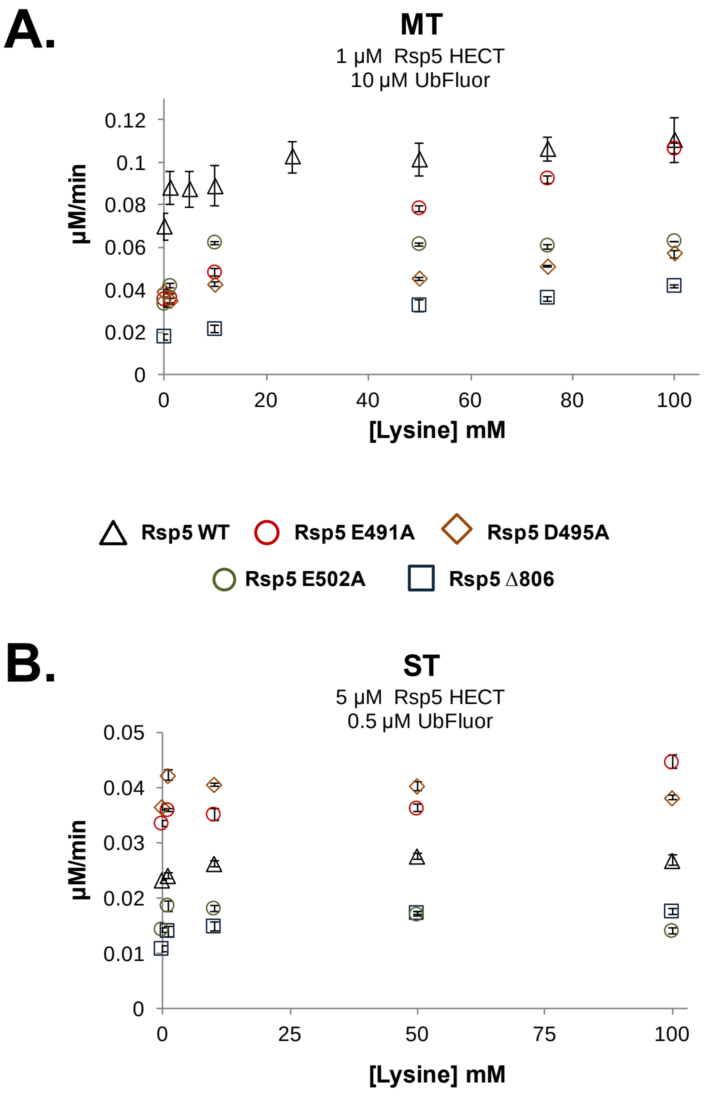

Figure S15. MT rates can increase in the presence of lysine, while ST rates are relatively unresponsive. (A) MT initial velocities were measured with fluorescence polarization. Reactions were run in 150 mM NaCl, 0.5 mM TCEP, 6 μM Tween-20, 50 mM HEPES pH 7.5 with the indicated amount of L-lysine. The indicated amount of L-lysine was added as part of a 10X buffer (pH was then adjusted to 7.5 for each 10X buffer) (B) ST initial velocities were measured with fluorescence polarization under the same buffer conditions described for (A). A background subtraction of raw polarization data was performed based on reactions without ligase at each concentration of L-lysine for (A) and (B). The plotted data are mean ± SEM for 3 separate reaction trials. Data for WT, E491A, and Δ806 are shown in Figure 5 of the main text.

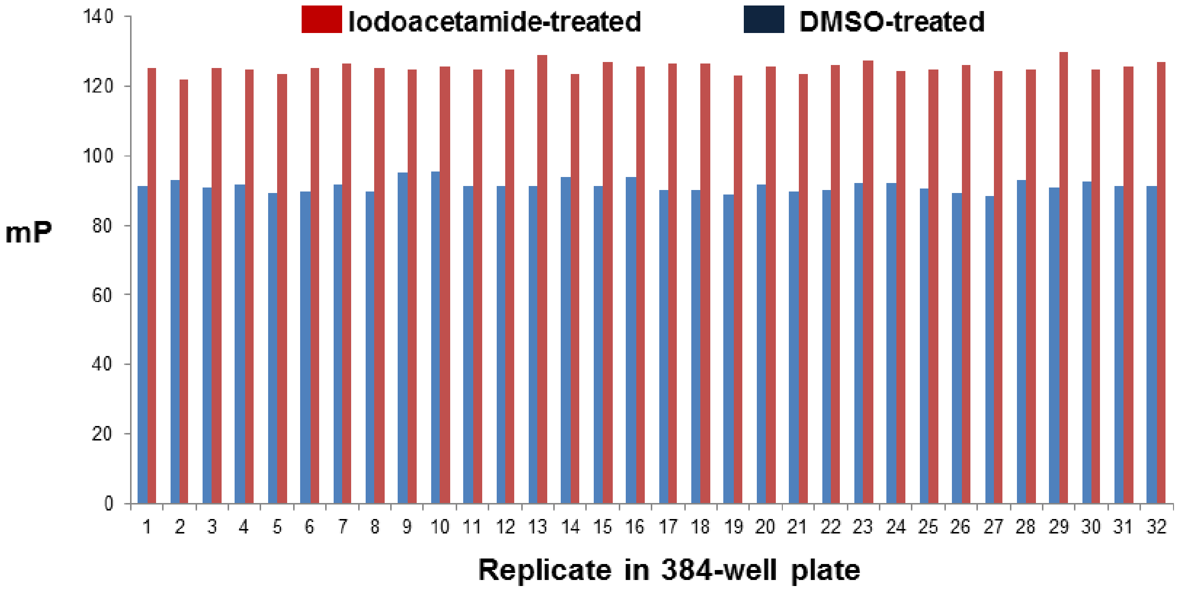

$$Z' = 1 - \frac{3 * (\sigma_{+} + \sigma_{-})}{< + > - < - >}$$

At 300 min, ***Z'*** = 0.72

Figure S16. UbFluor with Nedd4-1 HECT provides a Z' that is suitable for a drug discovery assay. Nedd4-1 HECT (0.5 µM) was incubated in a 384 well plate (Corning 3820) in 50 mM HEPES pH 7.5, 50 mM NaCl at 25 °C with DMSO (0.2%) or DMSO with iodoacetamide (1 mM) for 1 hour. UbFluor (5 µM) was then added and incubated with enzyme for 5 hours before endpoint fluorescence polarization readings were recorded with the Analyst GT plate reader (Molecular Devices). The Z' was calculated using the 32 replicates shown above in the bar graph. Each bar represents the measurement taken from a well.

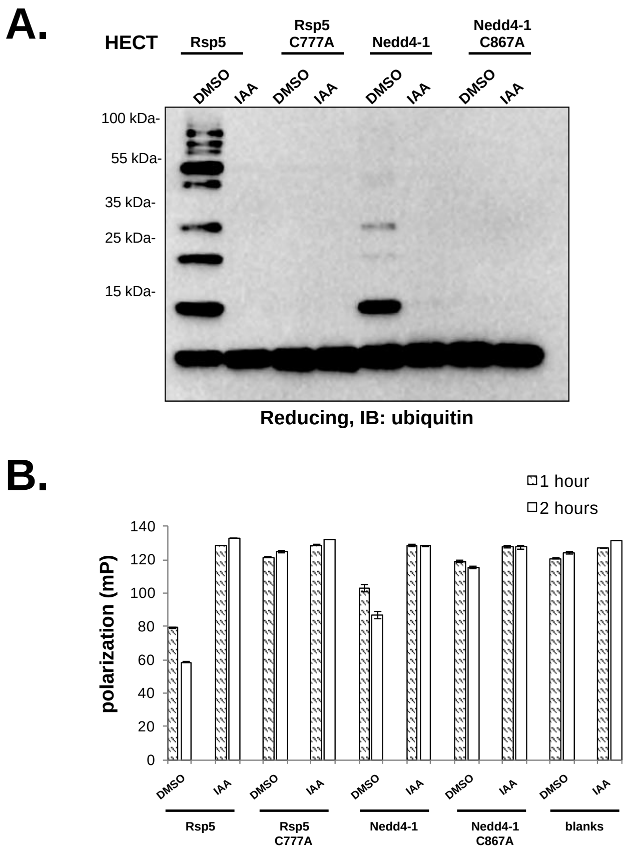

Figure S17. The catalytically inactive HECT domains Rsp5 C777A and Nedd4-1 C867A do not process UbFluor. (A) Ligase (1.0 μM) was incubated in 50 mM HEPES pH 7.5, 50 mM NaCl, 0.5 mM TCEP, 6 μM Tween-20 with DMSO (0.2%) or DMSO with iodoacetamide (IAA, 1 mM) for 1 hour. UbFluor (5 μM) was then added and incubated with ligase for two hours before quenching with reducing Laemmli buffer and resolving with SDS-PAGE. (B) Endpoint fluorescence polarization was measured for the same reactions of (A). Means ± SEM from 3 separate reaction trials are plotted.

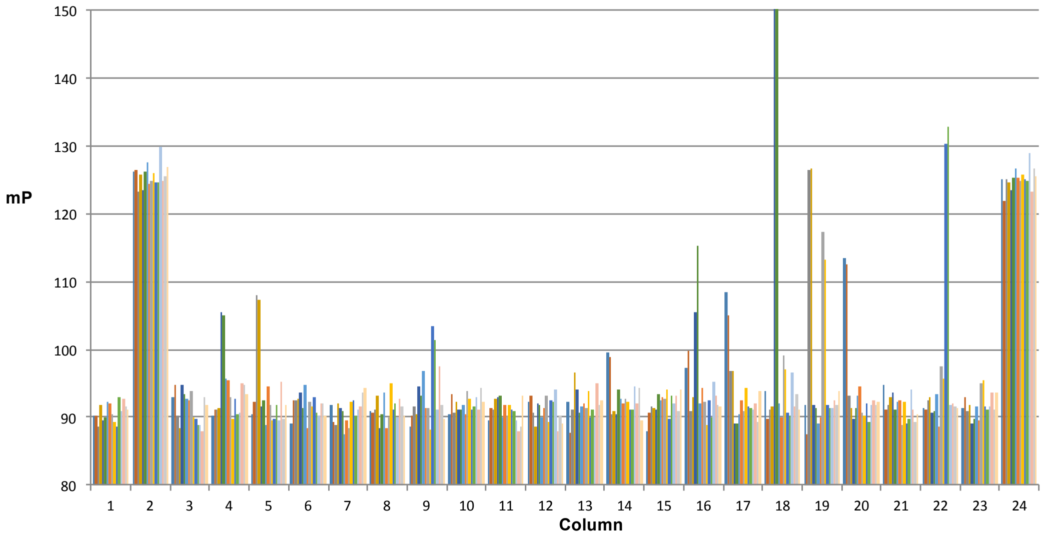

Figure S18. Hits in the screening assay are reproducible. Using the same screening format as Figure 6 of the main text, this experiment screened 160 small molecules from the MicroSource Spectrum Collection against Nedd4-1 HECT in duplicate, where duplicate molecules were dispensed to adjacent wells. The doublet peaks indicate how ‘hit’ molecules produce the same response in two separate wells. It is important to note that the MicroSource Spectrum Collection is a pilot library known to contain reactive compounds, thiol containing compounds, furans, redox cyclers, and compounds with nitro groups. As a result, this collection leads to an increased hit rate compared to the Maybridge plate in Figure 6C.

Nedd4-1 HECT (0.5 µM) was pre-incubated for 1 hour with small molecule (50 µM, 0.5% DMSO final concentration) in 50 mM HEPES 7.5, 150 mM NaCl, 6 µM Tween-20. UbFluor (5 µM final concentration) was then added and incubated with reaction solutions at 27 °C. Fluorescence polarization was read by the Analyst GT (Molecular Devices) at room temperature after 5 hours. Columns 1 and 23 had DMSO, and columns 2 and 24 had 1 mM iodoacetamide as controls.

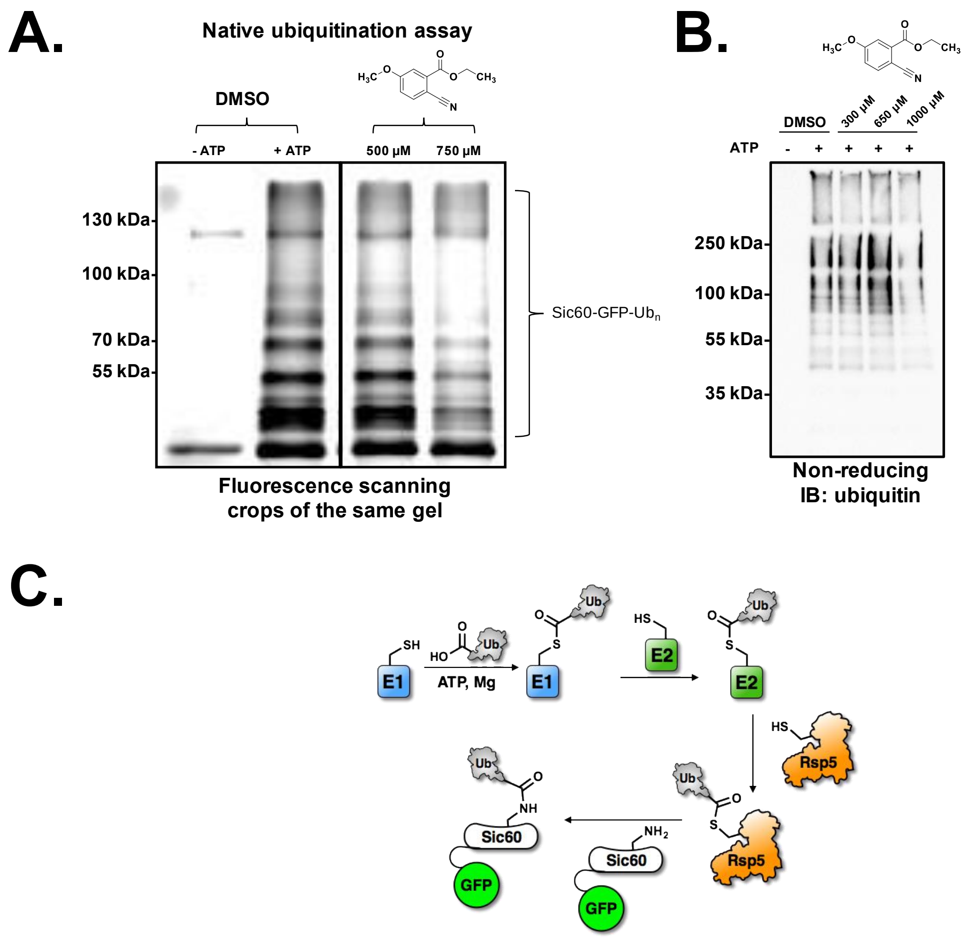

Figure S19. Freshly dissolved molecule from primary screen inhibits native ubiquitination of the fluorescent substrate Sic60-GFP. (A) 1.25 µM Nedd4-1 HECT was pre-incubated with 3% DMSO +/- small molecule for 1 hour at room temperature in 20 mM HEPES pH 7.5, 150 mM NaCl, 6 µM Tween-20. Ube1 (E1, 80 nM), UbcH7 (E2, 1.5 µM), Sic60-GFP (0.5 µM), ubiquitin (6 µM), 4 mM $MgCl_2$ were then added. Finally, 4 mM ATP was added to start the reactions. After 45 minutes at room temperature, the reactions were quenched with non-reducing 6x Laemmli buffer. The subsequent SDS-PAGE gel was imaged with the Typhoon 9400 scanner. (B) Same as (A) but run in 50 mM HEPES pH 7.5 and imaged by Western blot. (C) Cartoon description of Sic60 ubiquitination.

Supplementary Methods

Biochemical Procedures

General Information

UbE2W and UBE2K were purchased from R&D Systems. Sodium 2-mercaptoethanesulfonate (MESNa) and wild-type ubiquitin from bovine erythrocytes were purchased from Sigma-Aldrich. These purchased proteins and chemicals were used without further purification. In-gel fluorescence scanning was performed using the Typhoon 9400 (GE Healthcare). Fluorescence polarization was read with the Synergy4 plate reader (BioTek). All Coomassie images were obtained following incubation with InstantBlue stain (Expedeon). Anti-ubiquitin rabbit antibody was purchased from Cell Signaling Technology. Anti-K63-ubiquitin linkage antibody was purchased from Millipore. Goat Anti-Rabbit IgG (H+L)-HRP Conjugate antibody was purchased from Bio-Rad. GST-Rsp5 in pGEX-6p-1 in a pET3a vector was a gift from Prof. Andreas Matouschek, and Rsp5ΔWW in a pET30 vector was a gift from Prof. Linda Hicke. The original 3×FLAG-6×His-$Ub_{1\text{-}75}$ cloned into PTXB1 vector was a gift from Prof. David O. Morgan. Nedd4-1 plasmid was a gift form Prof. Simona Polo. All mutations were performed using the Quickchange II kit (Agilent Technologies). Protein concentrations were assessed by BioSpec-nano (Shimadzu) or Bradford assay (Bio-Rad). Fluorescently labelled ubiquitin for pulse-chase assays was purchased from LifeSensors.

Probe Synthesis

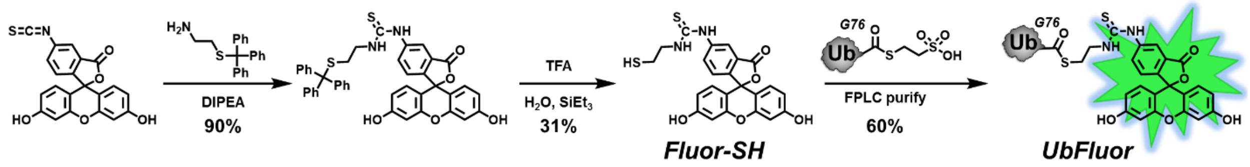

Synthesis of trityl-protected cysteamine. To a mixture of cysteamine hydrochloride (1 g, 8.8 mmol) and trifluoroacetic acid (1.3 mL, 17.6 mmol) in CH_2Cl_2 (30 mL), was added tritylchloride (2.4 g, 8.8 mmol). The reaction mixture was stirred for 16 hours at room temperature. The reaction was quenched by adding 1 M NaOH solution (20 mL) and the organic phase was diluted with CH_2Cl_2 (50 mL), washed with brine (20 mL) and dried over magnesium sulfate. The white crystalline solid was obtained from ether/n-pentane precipitation (455 mg, 1.42 mmol, 16 %

yield). ^{1}H NMR (400 MHz, $CDCl_3$) δ = 7.43 – 7.42 (d, 6H), 7.29 – 7.26 (t, 3H), 7.21 – 7.19 (t, 3H), 7.16 – 7.11 (m, 3H), 2.62 (t, 2H), 2.23 (t, 2H). ^{13}C NMR (125 MHz, $CDCl_3$) δ = 144.4, 129.8, 128.6, 127.4, 67.7, 36.9, 29.4.

Synthesis of trityl-protected-Fluor. To a mixture of trityl-protected cysteamine (330 mg, 1.0 mmol) and N,N-diisopropylethylamine (345.7 µl, 2.0 mmol) in DMF (5 mL), was added fluorescein isothiocyanate (Aldrich, isomer 1, 400 mg, 1.0 mmol). The reaction mixture was stirred at room temperature for 16 hours. The reaction was then stirred under an $N_{2(g)}$ stream to reduce the total volume to 1~2 mL. This concentrated solution was poured into 48 mL water (50 mM HEPES, pH6.5) in a 50 mL conical tube. The precipitated product was centrifuged at 4000 rpm for 10 min. The precipitate was dissolved in 2~3 mL of MeOH and passed through a silica plug with 50~60 mL ethyl acetate. It is further washed with ethyl acetate/methanol (8% MeOH) until all fluorescent material had eluted. The eluted material was concentrated under reduced pressure to give an orange powder (650 mg, 90 % yield). ^{1}H NMR (400 MHz, MeOD); δ = 8.17 (d, 1H), 7.74 (dd, 1H), 7.44 – 7.41 (m, 6H), 7.32 – 7.21 (d, 10H), 7.12 (d, 1 H), 6.69 (m, 3H), 6.53 (dd, 2H), 3.53 (t, 2H), 2.56 (t, 2H). ^{13}C NMR (125 MHz, MEOD) δ = 31.0, 42.6, 66.4, 102.1, 110.0, 112.2, 116.7, 124.2, 126.4, 127.6, 128.7, 128.9, 129.4, 130.5, 140.8, 144.8, 152.7, 159.9

Synthesis of Fluor-SH. To trityl-protected-Fluor (100 mg, 0.141 mmol) in a 20 mL glass scintillation vial was added TFA solution (1 mL trifluoroacetic acid with 25 µL triethylsilane and 25 µL water). The mixture was lightly vortexed for two hours at room temperature and then poured into diethyl ether (50 mL Et_2O in a conical tube). The precipitated product was centrifuged (4,000g, 10 min) to obtain an orange pellet (16.3 mg, 0.035 mmol, 83 % yield). To purify this product by HPLC, the pellet was dissolved in acetonitrile/water (3:7) with 0.1 % TFA (2 mL). A few drops of triethylamine were added to fully dissolve the solid. It was then purified with a gradient HPLC method (ramp from 30% -95% acetonitrile, column: Restek Pinnacle DB C18). The collected major peak was then lyophilized overnight to obtain an orange/red powder (20 mg, 31% yield). ^{1}H NMR (400 MHz, DMSO); δ = 8.22 (s, 1H), 7.74 (d, 1H), 7.19 (dd, 1H), 6.67 – 6.55 (m, 6H), 2.73 (t, 2H), 2.47 (t, 2H). ^{13}C NMR (125 MHz, DMSO); δ = 23.2, 47.3, 102.7, 110.2, 113.1, 121.1, 124.5, 129.5, 141.5, 147.7, 152.3, 159.9, 168.9

Ub~MES synthesis (E1 enzyme mediated synthesis)[5]

In a 50 mL conical tube, the following components were combined according to the given order of addition:

500 mM $NaPO_4$ pH 8.0 (10X buffer)	-- 2.336 mL (50 mM final concentration)
E1 enzyme UBE1	-- final concentration 0.25 μM)
ATP	-- 129 mg ATP disodium salt (final concentration 10 mM)
MESNa	-- 384 mg (final concentration 100 mM)
$MgCl_2$	-- 234 μL 1 M solution (final concentration 10 mM)
Ubiquitin	-- (20 mg (Sigma Aldrich), 100 μM final concentration)

Double distilled water was added to bring the final volume to 23.4 mL. The 50 mL conical tube was capped and then gently inverted a few times to mix the contents. It was then placed at 37 °C for 5 hours without any agitation. The solution was then removed from the incubator and concentrated from 23.4 mL to ~2 mL with an Amicon 3 kDa MWCO spin filter. The product was purified through a HiLoad Superdex 75 FPLC column equilibrated with Storage Buffer A (17 mg product obtained, 85% yield).

Ubiquitin-Fluor-SH conjugation

Lyophilized Fluor-SH was dissolved in a few milliliters of methanol and transferred to a tared 20 mL glass scintillation vial. The majority of methanol was removed with rotovap, and then the resulting solid was further dried under a $N_{2(g)}$ stream for 30 minutes. The net mass of Fluor-SH was then determined. Fluor-SH was then dissolved to 50 mM in DMSO/H_2O (1:1) with a minimal amount of saturated aqueous $NaHCO_3$ (~30 μL sat. $NaHCO_3$ solution to 20 mg Fluor-SH), which should provide full solubility and will change the color of the solution from yellow to red. It may also be necessary to briefly sonicate the solution in order to fully dissolve Fluor-SH. After setting up the reaction, leftover Fluor-SH solution can be stored at -20 °C. The reaction setup is below:

To each of 4 x 2 mL microcentrifuge tubes, the following reagents were added according to the listed order of addition (final total volume = 1111 uL) :

1 M HEPES pH 7.5	-- 111 μL (100 mM final concentration)
100 mM TCEP	-- 100 μL (9 mM final concentration)
6 M guanidinium chloride	-- 300 μL (1.62 M final concentration)
50 mM Fluor-SH	-- 300 μL (13.5 mM final concentration)
700 μM Ub-MESNa	-- 300 μL (189 μM final concentration)

After closing all 4 reaction tubes, they were covered in aluminium foil and set to lightly vortex for 120 minutes. At this point, Storage Buffer B (889 μL) was added to each tube to bring the total volume of each tube to 2.0 mL. The reaction solutions were added to a Slide-A-Lyzer Dialysis Cassette (3-12 mL size, 3,500 Da MWCO, Life Technologies) pre-hydrated in Storage Buffer B. Dialysis was performed in 2 L Storage Buffer 3 x 2 hours. Alternatively to dialysis, reaction solutions can be purified through a HiTrap Desalting Column (GE Healthcare, 5 mL size) prior to subsequent Superdex 75 FPLC purification (see below).

Dialyzed material was further purified through a HiLoad Superdex 75 FPLC column equilibrated with Storage Buffer B. UbFluor elutes around 120 mL total elution volume, while ubiquitin elutes around 90 mL. The UbFluor fractions were collected and concentrated with Amicon Ultra-15 Centrifugal Filter Units (to 50-100 μM). UbFluor was aliquoted, snap frozen in $N_{2(l)}$ and then stored at -80 °C (4.5 mg, 60% yield).

Fluorescence polarization measurement

Fluorescence polarization (FP) was measured using a Synergy4 (BioTek) fluorescence plate reader running Gen5 software (BioTek). All components for a given reaction were added to a 1.5 mL microcentrifuge tube, the appropriate volume of Rsp5 was placed on the wall of the tube, and then the reaction was initiated by centrifuging the tube at 10,000 *g* for seven seconds. The reaction solution was mixed by pipetting five times and then loaded (20 μL) to a 384-well plate (Corning 3820). Once all the samples were loaded to

the plate, the plate was spun at 1,500 *g* for eight seconds, and then added to the plate reader to record FP. All the reactions contained 150 mM NaCl, 6 μM Tween-20, 0.5 mM TCEP, 50 mM HEPES pH 7.5 unless otherwise stated. The dead time between spinning Rsp5 into solution and FP measurement was ~ 2 minutes. Typically, 4 – 8 reactions were analysed during a given reading. For single turnover assays, 4 concentrations of UbFluor (0.25, 0.50, 0.75, or 1.0 μM) with 5 μM of Rsp5 were analysed every 5 – 15 seconds. For multiple turnover assays, 4 concentrations of UbFluor (10, 12.5, 15, or 20 μM) with 1 μM of Rsp5 were analysed every 5 – 20 seconds.

High-throughput Screening assay

DMSO, iodoacetamide in DMSO, and DMSO stocks of screening molecules were dispensed (12.5 nL) to 384-well low volume, nonbinding surface plates (Corning 3820) with the Labcyte Echo 550. The Viafill liquid handler (Integra) was then used to dispense a solution of Nedd4-1 HECT in 50 mM HEPES pH 7.5, 150 mM NaCl, 6 μM Tween-20 to each well of the plates (15 μL). Plates were then centrifuged (2000 *g*, 15 seconds), lightly agitated (850 rpm on a horizontal platform, 5 minutes), and maintained at 27 °C for 1 hour. Then UbFluor in 50 mM HEPES pH 7.5, 150 mM NaCl, 6 μM Tween-20 was dispensed with the Viafill to each well of the plates (10 μL). The centrifugation and agitation steps were repeated, and plates were maintained at 27 °C. Fluorescence polarization was measured every subsequent hour with the Analyst GT (Molecular Devices). Final concentrations: Nedd4-1 HECT (0.5 μM), UbFluor (5 μM), small molecules (50 μM), iodoacetamide controls (1 mM), DMSO in all wells (0.5%).

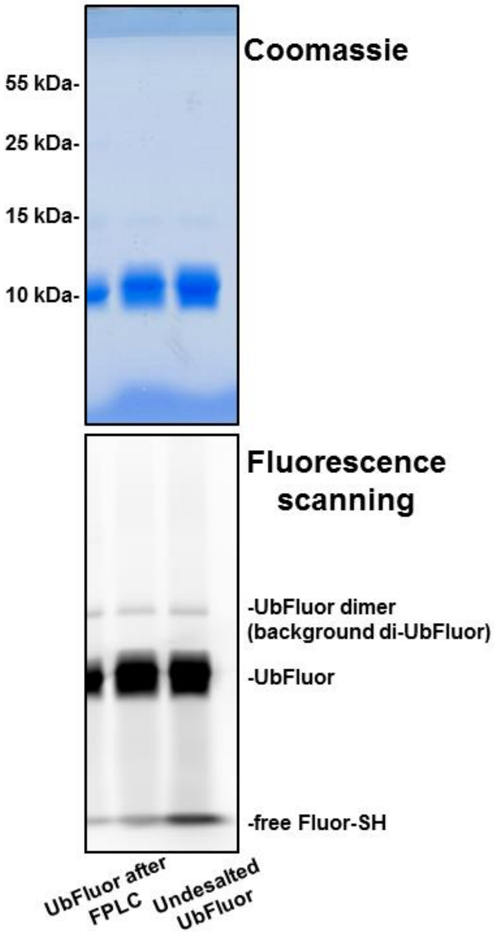

Figure S20. Gel-based analysis of UbFluor purification Samples were analysed by SDS-PAGE and imaged under Coomassie staining or fluorescence scanning. FPLC purification removes residual free fluorophore from the probe.

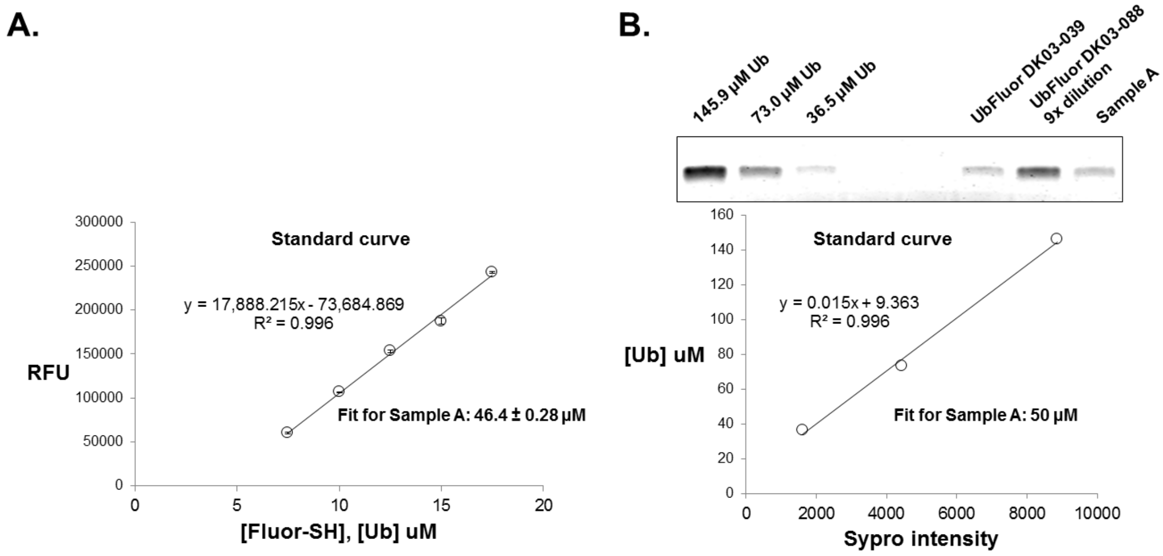

Figure S21. Methods to determine UbFluor concentration. (A.) Equal concentrations of Fluor-SH and ubiquitin were incubated in 1X PBS with 20 mM β-ME and analysed with a NanoDrop 3300 spectrofluorometer to make a standard curve. UbFluor (Sample A) was diluted with a four-fold excess of the PBS/β-ME buffer and was incubated at room temperature for 1 hour while the standard curve was generated. (B.) Ubiquitin from Sigma-Aldrich or UbFluor was diluted seven-fold in 1xPBS with 20 mM β-ME and incubated at 37 °C for 60 minutes. Samples were then mixed with 6X reducing Laemmli buffer and boiled for 5 minutes at 95 °C. The resulting solutions were then loaded (1833 ng for 1.25 mg/ml sample) to an 18% acrylamide gel (1.5 mm). Following SDS-PAGE, the gel was stained with Sypro Orange and imaged with the Typhoon 9400 scanner.

Mass spectra

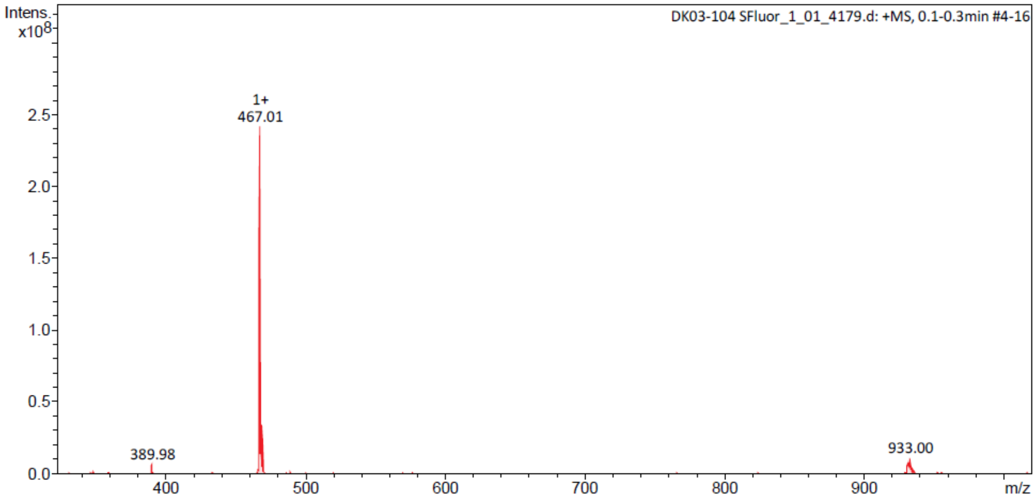

MS obtained for Fluor-SH on Bruker AmazonX Quadrupole Ion Trap mass spectrometer with Compass Software version 1.4 and MaxEnt deconvolution software: MS calcd for $C_{23}H_{18}N_2O_5S_2$: 466.53; Found: *m*/*z* 467.01

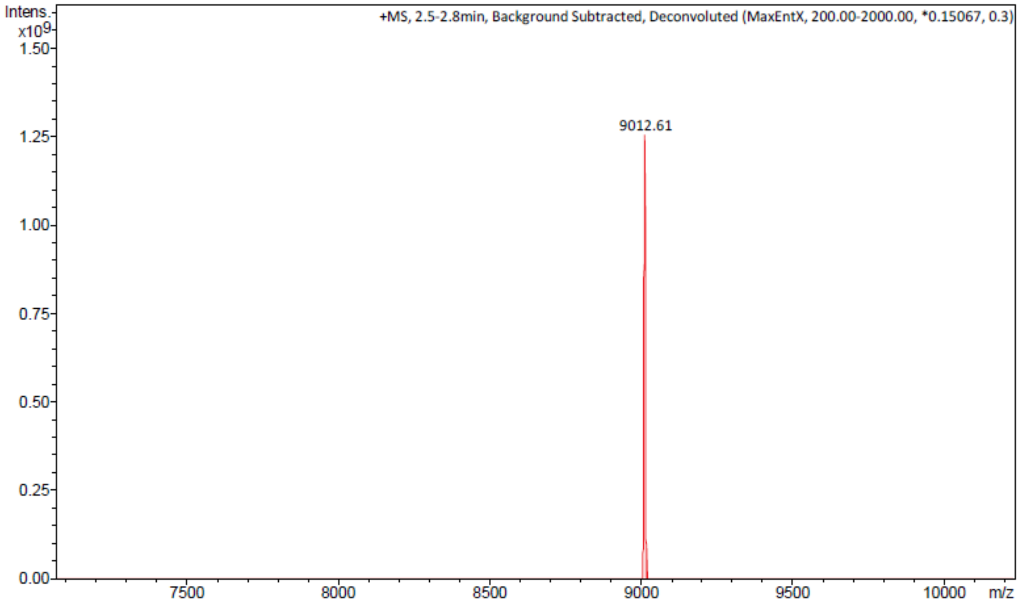

MS obtained for UbFluor on Bruker AmazonX Quadrupole Ion Trap mass spectrometer with Compass Software version 1.4 and MaxEnt deconvolution software: MS calculated for UbFluor: 9013.37

Protein Purification

Rsp5 HECT

E.Coli Rosetta (DE3) cells (Novagen) were transformed with GST-Rsp5 HECT (amino acids 421-809) cloned into the pGEX-6P-1 vector with 100 µg/ml ampicillin, 34 µg/ml chloramphenicol selection. Cells were grown at 37 °C until OD_{600} = 0.6, at which point they were incubated at 4 °C for 30 minutes. Expression was induced with IPTG (0.5 mM final concentration) at 18 °C overnight. Cells were then harvested by centrifugation (6000 *g*, 20 min, 4 °C), resuspended in PBS supplemented with Complete Mini Protease Inhibitor Cocktail (Roche), and DNAse I from bovine pancreas (10 µg/mL final concentration, Sigma Aldrich) and lysed by sonication. The lysate was cleared by centrifugation (18,*500* g, 40 min, 4 °C) and then filtered (0.45 µm). Filtered lysate was incubated with Glutathione Sepharose (1 mL bead slurry for 500 mL original culture, GE Healthcare) for 3 hours at 4 °C with rocking. The beads were then washed with PBS (4 x 5 mL), and then PCB (1 x 10 mL). Rsp5 was eluted following cleavage with PreScission Protease in PCB overnight at 4 °C. Rsp5 was purified through a HiLoad Superdex 75 FPLC column equilibrated with PCB. All Rsp5 mutants were prepared using the same experimental procedure.

UbcH7

GST-UbcH7 cloned into the pGEX-6P vector was transformed into *E.Coli* BL21 cells with 100 µg/ml ampicillin selection. UbcH7 was induced with 0.3 mM IPTG at 18 °C for 20 h (OD_{600} 3.0, 1 L terrific broth). Cell pellets (6000 *g*, 20 min, 4 °C) were resuspended in PBS with DTT (1 mM), $MgCl_2$ (10 mM), protease inhibitor (Roche COMPLETE), and DNAse I from bovine pancreas (10 µg/mL final concentration, Sigma Aldrich). Following sonication, lysate was cleared by centrifugation (18,*500 g*, 40 min, 4 °C). The supernatant was passed through a 0.45 µm syringe filter, and then added to glutathione agarose bead slurry (Pierce, 1.5 ml bead slurry per 1 L culture) that had been equilibrated with PBS supplemented with DTT (1 mM). After rocking at 4 °C for 12 h with lysate, the beads were washed with PBS supplemented with DTT (1 mM) (3 x 15 ml). UbcH7 was then cleaved from the beads by incubating with PreScission Protease (1 mg, GE Healthcare) in 1.5 mL PBS supplemented with DTT (1 mM) for 12 h at 4 °C on a rocker. Protein was then eluted with PBS supplemented with DTT (1 mM), concentrated to 150 µM and stored at -80 °C.

Preparation of UbcH5B

pGEX4T3-UbcH5B plasmid was generously provided to us from Dr. Arthur Haas. *E. coli* BL21 cells (Agilent) were transformed with the plasmid with 100 µg/ml ampicillin selection. Cells were grown at 37 °C until OD_{600} = 0.6, at which point expression was induced with IPTG (0.4 mM final concentration), and growth was continued for 2.5 hours. Cells were harvested by centrifugation (6000 *g*, 20 min, 4 °C), resuspended in lysis buffer (50 mM Tris, pH 7.5, 150 mM NaCl, 1 mM DTT, 4 °C), and lysed by sonication. The lysate was cleared by centrifugation (18,000 *g*, 40 min, 4 °C) and then filtered (0.45 µm). Filtered lysate was incubated with Glutathione Sepharose (3 mL bead slurry for 4 L original culture, GE Healthcare) for 2 hours at 4 °C with rocking. The beads were then washed with lysis buffer (3 x 15 mL). GST-UbcH5B was eluted with 50 mM Tris, pH 7.5, 1 M NaCl, 20 mM reduced glutathione. Fractions were pooled and dialyzed into 50 mM Tris, pH 7.5, 1 mM DTT, 4 °C. UbcH5B was then concentrated to less than 5 mL and cleaved with thrombin (GE Healthcare) before quenching with 4-(2-Aminoethyl)benzenesulfonyl fluoride hydrochloride (1 mM final concentration, Sigma Aldrich). UbcH5B was purified from uncleaved enzyme, GST, and thrombin via size exclusion chromatography using a Superdex 75 column (GE Healthcare).

Preparation of ΔWW Rsp5

Rsp5ΔWW (Figure S1) was transformed into *E. coli* Rosetta (DE3)pLysS with 100 µg/ml ampicillin, 34 µg/ml chloramphenicol selection (Novagen), and expression was induced with IPTG (0.2 mM final concentration) at 18 °C overnight. Cells were then harvested by centrifugation (6000 *g*, 20 min, 4 °C), resuspended in 6xHis purification Buffer A supplemented with DTT (1 mM), $MgCl_2$ (8 mM), and DNAse I from bovine pancreas (10 µg/ml, Sigma-Aldrich). Following sonication, the lysate was cleared with centrifugation (18,000 *g*, 30 min, 4 °C) and then incubated with TALON Metal Affinity Resin (Clontech; 2 ml slurry equilibrated with 6xHis purification Buffer A) for 1 hour at 4 °C with rocking. The resin was then washed with 6xHis purification Buffer A (20 ml) and 6xHis purification Buffer B (20 mL) before elution with 6xHis purification Buffer C (1 x 5 ml). The combined eluates were concentrated with

Amicon Ultra Centrifugal Filter Units (MWCO 30,000, Millipore) to 2 mL and then buffer exchanged to 400 mM NaCl, 100 mM HEPES pH 7.0, 1 mM TCEP (Zeba Spin Desalting Columns, Thermo). Rsp5ΔWW was concentrated to ~ 40 μM and stored at -80 °C.

Preparation of Nedd4-1 HECT

Nedd4-1 HECT domain (residues 938-1318) in a PGEX6P1 vector plasmid (GST-Nedd4-1 HECT) was transformed into *E. coli* BL21 cells (Novagen). 1L TB media containing 100μg/ml ampicillin was inoculated with 50 mL overnight cell culture and incubated at 37°C until OD reached ~3. Then, IPTG (1.0 mM final concentration) was added to the cell culture media at 18°C, followed by 16 hour incubation at the same temperature. Cells were then harvested (6000 *g*, 20 min, 4 °C) and lysed by sonication in PBS with protease inhibitors (Complete Mini Protease Inhibitor Cocktail, Roche). The lysate was cleared by centrifugation (18,000 *g*, 30 min, 4 °C) and then the supernatant was incubated with glutathione agarose beads (Pierce Biotechnology) for 1 hour at 4°C. The beads were washed three times with PBS and incubated with PreScission Protease (GE Healthcare) for 4h at 23°C to elute Nedd4-1 HECT domain (50mM HEPES, 150 mM NaCl, 0.1 mM EDTA 1mM DTT or 0.5 mM TCEP).

Preparation of Nedd4-2 HECT

We obtained a pET28-MHL plasmid containing the Nedd4-2 HECT domain without the last eight C-terminal residues (Addgene #25169). We added these residues by PCR reaction. The Nedd4-2 HECT domain (residues 594-975) was then expressed, purified and stored according to the procedure use for Rsp5ΔWW.

Preparation of WWP1 HECT

E. coli Rosetta (DE3) cells (Novagen) were transformed with a pET28 plasmid containing the WWP1 HECT domain (residues 546-922) with 15 μg/ml kanamycin, 34 μg/ml chloramphenicol selection. Cells were grown in LB media to OD_{600} = 0.7 before inducing expression with IPTG (1.0 mM final concentration) at 20 °C over six hours. Cells were then harvested by centrifugation (6000 *g*, 20 min, 4 °C) and resuspended in 50 mM Tris pH 8.0, 300 mM NaCl, 2 mM Imidazole, 1% Tween-20, 5% glycerol with protease inhibitors (Complete Protease Inhibitor Cocktail,

Roche). Following sonication, the lysate was cleared with centrifugation (18,000 *g*, 45 min, 4 °C), and then incubated with TALON Metal Affinity Resin (Clontech; 2 ml slurry equilibrated with the resuspension buffer) for 1 hour at 4 °C with rocking. The resin was then washed with 50 mM Tris pH 8.0, 300 mM NaCl, 2 mM imidazole, 1% Tween-20, 5% glycerol, 1 mM PMSF, 2 mM BME (1 x 10 ml), and then with 50 mM Tris pH 8.0, 300 mM NaCl, 20 mM Imidazole, 1% Tween-20, 5% glycerol, 1 mM PMSF, 2 mM BME (2 x 10 ml). His-tagged ligase was eluted with 50 mM Tris, 100 mM NaCl, 300 mM imidazole, 5% glycerol, pH 8.0 (2 x 1 ml) and then buffer exchanged to 50 mM Tris pH 8.0, 100 mM NaCl, 5% glycerol (Zeba Spin Desalting Columns, Thermo).

Preparation of ITCH HECT

E. coli BL21 (DE3) cells (Novagen) were transformed with a pET28-MHL plasmid containing the ITCH HECT domain (residues 544-903, Addgene #36197) with kanamycin selection. Cells were grown in terrific broth media to OD_{600} = 3 before inducing expression with IPTG (2.0 mM final concentration) at 15 °C over 16 hours. Cells were then harvested by centrifugation (6000 *g*, 20 min, 4 °C) and resuspended in 50 mM Tris pH 8.0, 500 mM NaCl, 5% glycerol, 2 mM imidazole, 0.1 mM PMSF, 10 mM BME with protease inhibitors (Complete Protease Inhibitor Cocktail, Roche). Following sonication, the lysate was cleared with centrifugation (18,000 *g*, 40 min, 4 °C), and then incubated with TALON Metal Affinity Resin (Clontech; 2 ml slurry equilibrated with the above resuspension buffer) for 2 hours at 4 °C with rocking. The resin was then washed with 50 mM Tris pH 8.0, 500 mM NaCl, 5% glycerol, 10 mM imidazole (4 x 15 ml). His-tagged ligase was eluted with 50 mM Tris pH 7.0, 250 mM NaCl, 5% glycerol, 200 mM imidazole (3 x 1.5 ml) and then buffer exchanged to 20 mM Tris pH 8.0, 500 mM NaCl, 5% glycerol, and 0.5 mM TCEP.

Preparation of E1 enzyme (mouse UBE1)

E. coli BL21 (DE3) cells (Novagen) were transformed with a pET-mE1 plasmid with 15 μg/ml kanamycin selection. Cells were grown in LB media to OD_{600} = 0.6 before inducing expression with IPTG (0.5 mM final concentration) at 16 °C over 20 hours. Cells were then harvested by centrifugation (6000 *g*, 20 min, 4 °C) and resuspended in 50 mM Tris pH 8.0, 150 mM NaCl, 0.1% Triton X-100, 1 mM DTT with protease inhibitors (Complete Protease EDTA-free

Inhibitor Cocktail, Roche). Following sonication, the lysate was cleared by centrifugation (18,000 *g*, 40 min, 4 °C), filtered (0.45 µm), and then incubated with Ni-NTA agarose (Qiagen; 2 ml slurry equilibrated with the resuspension buffer) for 2 hours at 4 °C with rocking. The resin was then washed with 50 mM $NaPO_4$ pH 8.0, 150 mM NaCl (5 x 15 ml). Enzyme was eluted with 50 mM $NaPO_4$ pH 8.0, 150 mM NaCl, 100 mM imidazole (3 x 3 ml) and then dialyzed to 20 mM HEPES pH 8.0, 100 mM NaCl, 1 mM DTT. Enzyme was purified through a HiLoad Superdex 75 FPLC column equilibrated with 20 mM HEPES pH 8.0, 100 mM NaCl, 1 mM DTT. Glycerol was added to 10%, and then UBE1 was snap frozen in $N_{2(l)}$ and stored at -80 °C.

Buffers

Storage Buffer A: 25 mM NaCl, 12.5 mM HEPES pH 6.7
Storage Buffer B: 250 mM NaCl, 12.5 mM HEPES pH 6.0
PCB (PreScission Cleavage Buffer): 50 mM HEPES pH 7.5, 150 mM NaCl, 0.1 mM EDTA, 0.5 mM TCEP.
PBS (phosphate-buffered saline): 137 mM NaCl, 2.7 mM KCl, 10 mM Na_2HPO_4, 1.8 mM KH_2PO_4
6×His purification Buffer A: $NaPO_4$ (50 mM, pH 7) and NaCl (300 mM).
6×His purification Buffer B: $NaPO_4$ (50 mM, pH 7), NaCl (300 mM) and imidazole (25 mM).
6×His purification Buffer C: $NaPO_4$ (50 mM, pH 7), NaCl (300 mM) and imidazole (250 mM).

Supporting References

1. S. Park, D. T. Krist and A. V. Statsyuk, *Chem Sci*, 2015, **6**, 1770-1779.
2. H. B. Kamadurai, J. Souphron, D. C. Scott, D. M. Duda, D. J. Miller, D. Stringer, R. C. Piper and B. A. Schulman, *Mol Cell*, 2009, **36**, 1095-1102.
3. H. C. Kim, A. M. Steffen, M. L. Oldham, J. Chen and J. M. Huibregtse, *EMBO Rep*, 2011, **12**, 334-341.
4. Y. Sheng, J. H. Hong, R. Doherty, T. Srikumar, J. Shloush, G. V. Avvakumov, J. R. Walker, S. Xue, D. Neculai, J. W. Wan, S. K. Kim, C. H. Arrowsmith, B. Raught and S. Dhe-Paganon, *Mol Cell Proteomics*, 2012, **11**, 329-341.
5. F. El Oualid, R. Merkx, R. Ekkebus, D. S. Hameed, J. J. Smit, A. de Jong, H. Hilkmann, T. K. Sixma and H. Ovaa, *Angew Chem Int Ed Engl*, 2010, **49**, 10149-10153.
